# Evaluating allopolyploid origins in strawberries (*Fragaria*) using haplotypes generated from target capture sequencing

**DOI:** 10.1186/s12862-017-1019-7

**Published:** 2017-08-04

**Authors:** Olga K. Kamneva, John Syring, Aaron Liston, Noah A. Rosenberg

**Affiliations:** 1 Department of Biology, Stanford University, 371 Serra Mall, Stanford, CA 94305 USA; 2 Department of Biology, Linfield College, McMinnville, OR 97128 USA; 3 Department of Botany and Plant Pathology, Oregon State University, Corvallis, OR 97331 USA

**Keywords:** Gene trees, Haplotypes, Hybridization, Polyploidy, Species networks, Strawberry, Target capture sequencing

## Abstract

**Background:**

Hybridization is observed in many eukaryotic lineages and can lead to the formation of polyploid species. The study of hybridization and polyploidization faces challenges both in data generation and in accounting for population-level phenomena such as coalescence processes in phylogenetic analysis. Genus *Fragaria* is one example of a set of plant taxa in which a range of ploidy levels is observed across species, but phylogenetic origins are unknown.

**Results:**

Here, using 20 diploid and polyploid *Fragaria* species, we combine approaches from NGS data analysis and phylogenetics to infer evolutionary origins of polyploid strawberries, taking into account coalescence processes. We generate haplotype sequences for 257 low-copy nuclear markers assembled from Illumina target capture sequence data. We then identify putative hybridization events by analyzing gene tree topologies, and further test predicted hybridizations in a coalescence framework. This approach confirms the allopolyploid ancestry of *F. chiloensis* and *F. virginiana,* and provides new allopolyploid ancestry hypotheses for *F. iturupensis, F. moschata*, and *F. orientalis*. Evidence of gene flow between diploids *F. bucharica* and *F. vesca* is also detected, suggesting that it might be appropriate to consider these groups as conspecifics.

**Conclusions:**

This study is one of the first in which target capture sequencing followed by computational deconvolution of individual haplotypes is used for tracing origins of polyploid taxa. The study also provides new perspectives on the evolutionary history of *Fragaria.*

**Electronic supplementary material:**

The online version of this article (doi:10.1186/s12862-017-1019-7) contains supplementary material, which is available to authorized users.

## Background

Polyploidy is a property of an organism in which cells contain more than two complete sets of chromosomes. It has been an important evolutionary force shaping the process of diversification over the full history of eukaryotic life [1–3]. Evidence of polyploidization has been documented along a continuum from ancient speciations dating to 300–500 Mya [4, 5] to recent events of the last 100 years [2, 6–10]. Rates of polyploid speciation [11, 12], evolutionary consequences of polyploid formation [3, 13–15], factors promoting polyploidy [16, 17], and genomic modifications taking place after polyploidization [18–21] have been of recent and ongoing interest.

Polyploidy is often associated with plant evolution in particular, as all angiosperms are recognized to have a paleopolyploid ancestry [5], and ~15% of speciation events in this group are associated with polyploidization [11]. However, hundreds of examples of polyploid animals, invertebrate and vertebrate, are also known [13, 22]. Polyploid organisms are broadly classified as either auto- or allopolyploid in origin [23–25]. Allopolyploidy is defined by hybridization between two separate species that results in a polyploid genome, whereas autopolyploidy results from within-species genome duplication. Evidence of allopolyploid speciation has been documented for many eukaryotes, including a substantial number of plants [6, 26–31].

Hybridizations that result in polyploid speciation combine homeologous chromosomes from two or more distinct lineages, each with its own evolutionary history [2, 32]. Consequently, accurate identification of hybrid progenitors has critical importance to phylogenetic reconstruction. Many studies have used incongruence between nuclear (predominantly ribosomal DNA) and cytoplasmic genomes (chloroplasts or mitochondria) as evidence of hybrid origins of a species [32–37]. This approach, however, is limited to cases in which ribosomal DNA and genes encoded in chloroplast or mitochondrial genomes accurately record alternative evolutionary histories [38]. Additionally, ribosomal DNA is affected by concerted evolution that promotes homogeneity between paralogs inherited from distinct parental species, making the detection of separate ancestral genomes difficult [39, 40].

Low-copy nuclear genes are more likely to have retained homeologs—homologous genes inherited from the hybridizing parents—for each ancestor [39, 40]. Such loci record distinct ancestral histories and are therefore informative in reconstructing hybridization events. Nuclear loci are a rich source of phylogenetic markers. They are largely unlinked, span a range of substitution rates, and are less prone to concerted evolution than ribosomal DNA [41]. However, obtaining haplotype-specific sequences representing all copies of a given marker is challenging, especially for polyploids [42]. As a result, many phylogenetic studies still use consensus assembly of single sequences, even for potentially allopolyploid species [43, 44].

### Haplotypes in polyploids

A variety of approaches exist to produce haplotype data for polyploids. The most frequently employed methods separate gene copies *prior* to sequencing [45–49]. Strategies for haplotype or homeolog isolation include extracting DNA from available haploid material [50], obtaining inbred lines [51, 52], developing homeolog-specific primers [41, 51, 53–56], utilizing bacterial cloning [57], running single molecule PCR (smPCR) [41, 58], and employing single-strand conformation polymorphism (SSCP) gels to isolate homeologs [59].

Each of these methods is associated with significant challenges. Bacterial cloning, the most commonly employed method, is expensive and labor intensive, and involves the risk of missing alleles, selecting alleles generated through PCR error [60], or bias towards shorter inserts. More generally, PCR-based methods are either labor intensive, likely to result in large amounts of missing data, or both.

Notably, next-generation sequencing (NGS) provides new ways of computationally separating individual haplotypes, as individual reads generated by NGS are derived from a single DNA molecule and hence are haplotype-specific by nature [61]. Extending beyond similar approaches for diploids [62, 63], recent methods allow for haplotype assembly from NGS reads in polyploids [64–66]. For example, HapCompass, the tool we use in this study, phases haplotypes during assembly by using co-occurrence within NGS reads of alleles at different sites along a chromosome. To generate haplotypic data for phylogenetic inference on hybrids, target capture [67] and NGS followed by computational haplotype assembly circumvents many of the problems of molecular isolation approaches.

### Identifying hybridization events involved in polyploid formation

In the subsequent phylogenetic analysis of haplotype-specific sequences obtained from a set of taxa, each gene tree is multilabeled, and contains more than one sequence per taxon. Such multilabeled trees can be combined into species networks [19] using, for instance, a consensus cluster-network method [41, 53, 68–70].

However, other evolutionary processes besides hybridization, such as incomplete lineage sorting (ILS), can also lead to incongruence between gene genealogies [71]. In closely related species—exactly those species that tend to hybridize—ILS leads to a pattern similar to that generated by hybridization in the discordance between gene trees [72]. This issue renders consensus approaches to reconstructing hybridization histories of closely related species problematic. Therefore, methods have been proposed for accounting for ILS while reconstructing ancestral hybridization events [73–77]. Although such methods do provide a conceptual advance, they can be limited either by their computational burden or by restrictive modeling assumptions. For instance, it takes a long time to run likelihood-based inference in PhyloNet when many taxa and a complex evolutionary scenario are considered. STEMhy only operates on a set of four taxa: a hybrid, two sister parental taxa, and an outgroup.

One approach for circumventing these difficulties is to identify candidate hybridization events via a consensus summary of a set of multilabeled gene trees and to further test every candidate hybridization in an ILS-aware likelihood framework [74, 78] against a scenario with no hybridization. The idea is that a consensus approach might report erroneous hybridization events when ILS is not specifically considered, but that further ILS-aware tests would not support them. Here, we utilize this strategy with a large number of multilabeled gene trees generated for a set of *Fragaria* (strawberry) species.

### Fragaria

*Fragaria* is an emerging model system in evolution and ecology and is an archetype for the study of allopolyploid speciation (reviewed in [79]). This contribution follows in a long line of crop plants that have yielded critical insight into plant genomics and evolution [21, 80].

The genus is relatively young, with extant species estimated to have last shared a common ancestor 1.0–4.1 million years ago [81]. Wild *Fragaria* have a northern hemisphere distribution that includes a single species in southern South America. The genus has a center of diversity in China, and strawberries are found wild on all continents except Australia and Antarctica. Currently, 22 species of strawberry are accepted. Ten of these are polyploids, with a complete series of even ploidy levels ranging from tetraploid to decaploid. Though some uncertainty exists, the polyploids have been treated as having hybrid rather than autopolyploid ancestry.

Prior phylogenetic work in *Fragaria* has been based on morphological and cytological characteristics [82], chloroplast sequence data including whole cpDNA genomes [81, 83], and high- and low-copy nuclear regions on the order of one to two loci [83–85]. Collectively, these studies highlight both areas of high certainty (e.g. the presence of a “vesca” and a “China” clade) as well as poorly resolved relationships (e.g. the placement of *F. iinumae, F. nilgerrensis,* and *F. viridis*), including the precise parental heritage of most of the polyploids [30]. The China clade includes 4 diploid (*F. chinensis, F. daltoniana, F. nubicola, F. pentaphylla*) and 4 tetraploid species (*F. corymbosa, F. gracilis, F. moupinensis, F. tibetica*) endemic to China and adjacent Himalayan countries and one diploid species endemic to Japan (*F. nipponica*) [79]. The vesca clade includes 9 species: the closely related diploids *F. bucharica, F. mandshurica*, and *F. vesca,* the tetraploid *F. orientalis,* the hexaploid *F. moschata*, the octoploids *F. chiloensis* and *F. virginiana*, and the decaploids *F. cascadensis* and *F. iturupensis*. The cultivated and naturally occurring hybrids of *F. chiloensis* and *F. virginiana* are called *F. ×ananassa*.

Here, we examine allopolyploid speciation in strawberries in a phylogenetic framework using target capture data. Our dataset includes nearly complete taxon sampling: 20 species and subspecies covering all assumed diploid progenitors (*F. hayatai,* a close relative of *F. nilgerrensis* endemic to Taiwan, was not sampled) and all polyploids except the recently described *F. cascadensis* [86]. We use an extensive set of 257 low-copy nuclear loci and reconstruct haplotypes using HapCompass, the first tool allowing direct haplotype assembly for polyploids [87]. We first determine a “background” species tree on which hybridization events can occur. We then propose hybridizations from gene tree topologies, further testing each such event in an ILS-informed likelihood framework in STEMhy [88] and PhyloNet [78]. We apply this strategy to obtain insights into the polyploid origins and evolution of strawberry species.

## Methods

**Fig. 1:**
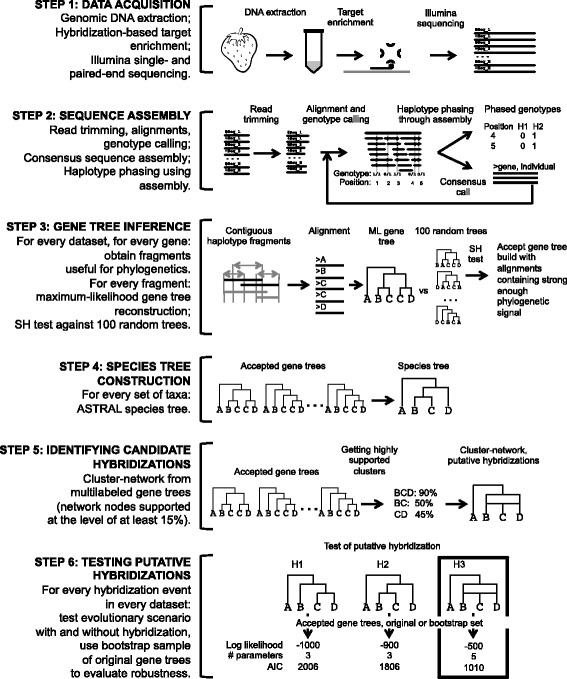
Overview of the analysis. *Step 1* of the workflow illustrates DNA extraction and target capture sequencing. *Step 2* includes standard NGS data clean - up steps such as read trimming, mapping to reference, and variant calling performed as described in the “Sequence and haplotype assembly” subsection of the methods. Haplotypes were phased using assembly with the HapCompass program, which utilizes the fact that alleles of different markers will co-occur in NGS reads if they are present within same the haplotypes. *Step 3* of the workflow includes a procedure for identifying regions of contiguous haplotype assembly across individuals represented by haplotypes within the alignments. Then standard steps in phylogeny reconstructions are included to obtain gene phylogenies. However, since some alignments were short with few informative sites, we used the SH test to evaluate if alignments do contain detectable phylogenetic signal. *Step 4* of this workflow illustrates inference of a species tree from a set of gene trees. *Step 5* depicts the summarizing of gene trees into consensus network. Step 6 shows the testing of candidate hybridizations in an ILS-aware framework

The workflow of our data analysis is outlined in Fig. 1 and is summarized here, with greater detail provided in subsequent sections. Illumina sequencing reads were obtained via target capture for 1419 exons corresponding to 257 genes from 20 *Fragaria* species and *Drymocallis glandulosa*, an outgroup species. Sequencing data were analyzed in two rounds to obtain unphased genotypes and consensus assemblies. In the first round, the *F. vesca* genome was used as a reference and Illumina reads were mapped to the reference to obtain an individual-specific consensus sequence assembly for every gene. In the second round, reads were aligned to the individual-specific reference sequences (generated in the first round of assembly) and final unphased genotypes were generated. Using linkage information captured by NGS reads, unphased genotypes were phased to obtain haplotypes for every individual at every gene. Assembled sequences were used to construct multilabeled gene trees for each assembled block of sequence. The topologies of the multilabeled gene trees were summarized in a consensus format to identify putative hybridization events leading to the formation of polyploid species. Lastly, putative hybridization events were tested in an ILS-aware framework to confirm some of the candidate hybridizations.

### Samples and sequencing

In this step of the analysis, plant tissue from 54 individuals was used to obtain sequencing reads for 257 phylogenetic markers for each individual (Fig. 1, step 1).

#### DNA samples and probe design

Fifty-three individuals of 20 species of *Fragaria*, representing the taxonomic diversity of the genus, and one individual from an outgroup taxon, *Drymocallis glandulosa*, were included in the analysis (Additional file 1: Table S1). *F. vesca* was intensively sampled because it is the most widespread species and has been subdivided into multiple subspecies. Diploid genomic DNA was extracted from leaf tissue using the FastDNA Kit® (Qbiogene, Irvine, CA, USA). A total of 50–100 mg of tissue was used for extractions. In cases in which collections were acquired as seed, samples were grown in a Percival growth chamber following standard germination protocols for *Fragaria*, and leaf tissue was collected as it became available.

Hybridization probes used in the targeted capture of 1419 exons were developed following Liston [89]. Briefly, putative single-copy genes were identified in the strawberry genome using BLAT [90]. In order to obtain sufficient phylogenetic resolution, each gene was required to contain at least 960 bp of sequence. To maximize target capture, all exons <80 bp long, with GC content <30% or >70%, and with >90% sequence similarity to annotated repetitive DNA in the genome were removed. Each retained exon was compared to the full set of retained exons; any exon with >90% sequence similarity to another target exon in the reference *F. vesca* genome was excluded. These steps produced 1419 exons from 257 genes, which were then used for final probe design (MycroArray, Ann Arbor, MI). For exons 80–120 bp long, a single oligonucleotide probe was used (1X tiling), and for exons >120 bp long, a 50% overlap (1.5X tiling) was used. The average targeted locus was 1744 bp long in 1–20 exons (mean = 5.5 exons), with average GC content 44.1%. Targeted sequence totaled 448,163 bp, with genes dispersed throughout the *F. vesca* genome. Over 95% (245) of the genes have a putative *Arabidopsis* ortholog, and 182 have a known molecular function.

#### Library preparation, solution hybridization, and sequencing

Library construction, solution hybridization, and sequencing followed Weitemier et al. [67]. Illumina sequencing libraries were prepared with NEBnext reagents (New England Biolabs, Ipswich, MA), Bioo index adapters (Bioo Scientific, Austin, TX), and 200 bp inserts size-selected from agarose gels.

The enriched pools were sequenced on two lanes of 101-bp single-end reads and 2 lanes of 101-bp paired-end reads with the Illumina HiSeq 2000 and one lane of 250-bp paired-end reads with the Illumina MiSeq by the Center for Genome Research and Biocomputing at Oregon State University. Some samples were sequenced more than once due to low read output. Diploids were sequenced using 101-bp single- and paired-end reads, and polyploids were sequenced using 250-bp and 101-bp paired-end reads. Base calling and sample demultiplexing followed standard Illumina workflows. Sequencing reads were deposited in the National Center for Biotechnology Information Sequence Read Archive.

### Sequence and haplotype assembly

In this step of the analysis, raw sequencing reads generated for 257 nuclear genes in 54 individuals and contigs from *F. vesca* were used as the input. The end products for every individual across each of the 257 genes include: (1) consensus sequence; (2) a set of unphased variants; and (3) a set of phased variants (Fig. 1, step 2). An online tutorial for this section of the analysis is also available [91].

To prepare references for read mapping, a blastn search was used to map all 1419 exons [89] onto *F. vesca* scaffolds using genome assembly v.1.1 [92]. Manual adjustment for exons not mapped over their entire length was conducted as needed. Contig sequences were extracted for every gene, including all exons, introns, and an additional 300 bases upstream of the first exon and 300 bases downstream of the last exon, and were used as reference sequences for read mapping (Additional file 2). Locus coordinates appear in Additional file 3: Table S2. The iterative assembly process is outlined here and depicted graphically in Additional file 4: Figure S1. In general, we follow the “best practices” workflow that appears on the GATK website.

1. Reference sequences were indexed with samtools-0.1.19, and a dictionary of the reference sequences was created using picard-tools-1.96 [93]. Either sequence from the whole-genome assembly of *F. vesca*, obtained as described in the previous paragraph, or individual-specific consensus sequences obtained in step 4 of the first iteration of this pipeline were used.
2. Raw sequence reads from Illumina sequencing were trimmed with Trimmomatic-0.32 [94], removing regions of poor quality and adapter sequences.
3. Trimmed reads were mapped to the reference sequences using bwa-0.7.5a [95]. An alignment was created in sam and converted to bam format, followed by sorting and indexing using samtools. Sequencing pools generated from the same individuals across multiple Illumina runs were merged, sorted, and indexed again; duplicate reads were removed from the merged files with samtools. Reads were realigned around indels using GenomeAnalysisTK-2.6-5-gba531bd [63]. Genotypes were called with GATK using bam files from the previous step and setting ploidy for each individual. Low-quality genotypes were excluded (QUAL < 50 and QD, “Variant Confidence/Quality by Depth”, <2). Among high-quality genotypes, we retained only those with read depth > 4; for heterozygous variants, we required at least 4 reads to have the minor allele. Subsequently, base-quality scores were recalibrated; the calibrated bam file was obtained for every individual and was used to call genotypes with GATK again. This step produced unphased genotypes used in further analysis.
4. Genetic variants obtained in the previous step were supplied to GATK to generate consensus sequences for every gene for every individual. These sequences were used as new references for every individual, and steps 1–3 were repeated with these individual-specific references. Unphased variants obtained in step 3 in the second iteration were supplied to step 7.
5. Mpileup and view commands from samtools were used to summarize bam files to obtain site-specific coverage information for every locus for every individual. Positions with fewer than 3 aligned reads were considered to have low coverage.
6. For every gene, in every individual, the fraction of positions with low coverage was calculated, and was then normalized by subtracting the mean and dividing by the standard deviation calculated across individuals for that gene (Additional file 4: Figure S2). Normalized values were averaged across genes within each individual. Fourteen individuals from seven species had a value of 0.25 or larger and were excluded from further analysis (Additional file 4: Figure S2).
7. Haplotype assembly was carried out using HapCompass v0.6.2 [87]. Calibrated bam files and vcf files obtained in step 3 in the second iteration of the pipeline were used to phase genotypes using linkage information within read data, setting the ploidy for each individual. This step yielded phased genotypes in haplotype assembly blocks for every gene for every individual.

### Gene tree reconstruction

In this step, individual-specific haplotype and consensus sequences were used to produce gene trees for each alignment found suitable for phylogenetic reconstruction (Fig. 1, step 3). We also developed an online tutorial that describes each step of this section [96].

#### Constructing datasets

**Fig. 2:**
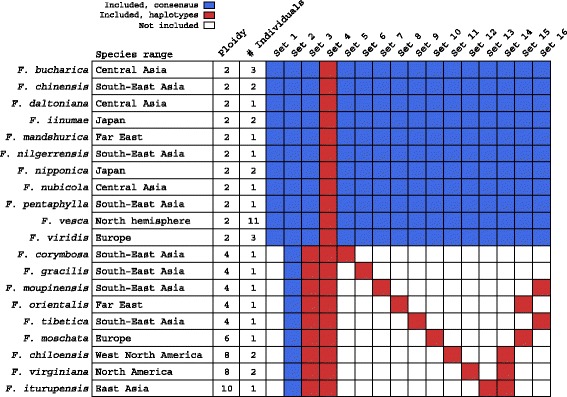
Taxonomic and sequence composition of different datasets used for phylogenetic analysis. Sampled species, their geographic range, ploidy level, the number of individuals included from each species, and taxonomic sampling in every dataset are shown. For each dataset, the total number of aligned base pairs is shown as well. Consensus sequences (or haplotypes in the case of dataset 5) from one individual from a diploid outgroup species, *Drymocallis glandulosa*, were included in every dataset and were used to root gene trees

The sets of taxa used in each round of analysis and the choices of consensus versus haplotype assembly appear in Fig. 2. Alignments were determined to be useful for phylogenetic inference if they were longer than 500 bp (600 bp for dataset 5 as this dataset contains the largest number of sequences per alignment because all individuals are represented by haplotypes). In dataset 5, in which all of the genomes were included as haplotypes, only 69 alignments exceeded 600 bp in length, summing to 40,736 aligned base pairs. This value is 10-fold fewer than the 423,081 aligned base pairs obtained for dataset 3, where all individuals are represented by consensus sequence, and no positions were lost due to gaps in haplotype phasing. Dataset 5 yielded a species tree with low bootstrap values. As expected in cases of allopolyploid origin of polyploid species, when we examined distances between haplotypes derived from the same individuals, we found that haplotypes from diploid individuals were more closely related to each other than were haplotypes from polyploid individuals. This was particularly true for individuals from the octoploid species *F. virginiana* and *F. chiloensis* and the decaploid lineage *F. iturupensis* (Additional file 4: Figure S3). This pattern suggested that haplotypes from diploids would likely group together in the phylogenetic analysis and would not provide additional information on the ancestry of polyploids, while at the same time introducing additional breaks in haplotype assembly blocks across individuals. Because the main goal was to infer the origin of polyploid species, we chose to represent the diploid individuals as consensus sequences in dataset 4 (Fig. 2). This choice substantially increased the number of alignments that were useful for phylogenetic analysis (from 69 in dataset 5 to 282 in dataset 4).

However, even when using consensus sequences for diploids and haplotypes for polyploids (dataset 4), well-resolved gene trees were not obtained. We therefore evaluated polyploid progenitors using partial taxon sets. These sets included the consensus sequence for all diploid species along with one polyploid species represented by haplotypes (Fig. 2, datasets 6–14). Subsequent analyses considered each dataset individually.

#### Generating alignments

Here we describe how fragments of genes with continuous haplotype assembly were identified, and how alignments were generated for one generic case. References generated as a consensus for every individual in a dataset for every gene were aligned to each other using MAFFT v7.164b [97] (linsi --op 1.53 --ep 1 command-line options). Next, for each gene, haplotype assembly blocks from individuals included as haplotypes (e.g., in dataset 12, *F. chiloensis* individuals) were mapped onto the consensus alignment using custom scripts with alignments and coordinates of haplotype assembly blocks as the inputs. Regions with continuous haplotype assembly were determined as follows, first from left to right and then from right to left (pseudo code in Additional files 4: Figures S4 and S5).

Working from left to right and starting from “position” 1 as the left (5′) border of the alignment fragment, all haplotypes covering this position were identified and were preserved in list “A”. In the case of the very beginning of the gene (or the very end of it), there can only be one haplotype block per individual covering the “position”. However, once the procedure moves to the next iteration, two haplotype assembly blocks could potentially cover this position in some individuals (for instance, iteration 2 of the procedure highlighted in Additional file 4: Figure S5). The right (3′) border was determined as the right-most position covered by at least one of the haplotypes from each individual from list A; these haplotypes were saved for further consideration. As an example, in Additional file 4: Figure S5 iteration 2, the individual depicted in gray would only have one haplotype remaining in list “A”, the one that reaches the end of the gene. The rest of the haplotypes were removed from list A, and the right border position was recorded. The left border was then changed to the left-most position covered by at least one of the haplotypes from list A from each individual. The utilized fragment was determined by the left and right border positions and haplotype names. The new “position” was set to the location of the right border of the previous fragment plus 1, and the process was repeated until the end of the alignment.

Haplotype fragments were selected for phylogenetic analysis on the basis of length, from longest to shortest. Because generated fragments can overlap with each other, the longest fragment available was selected first, all overlapping fragments were excluded, and the process was repeated until no fragments of suitable length remained. Haplotypes from the same fragment were aligned to each other and to consensus sequences from diploids for datasets 4 and 6–14, using MAFFT as above (Fig. 2). In each alignment, flanking regions of alignments not covered by haplotypes were trimmed and regions with insufficient sequence coverage were removed.

#### Building and testing gene trees

For every dataset (Fig. 2), nonoverlapping aligned fragments of appropriate length were used for phylogenetic inference. Phylogenetic trees were reconstructed from every fragment using PhyML [98] run with 100 non-parametric bootstraps to evaluate clade support, implementing the best-fitting evolutionary model as determined by jModelTest 2.1.7.2 [99]. Every gene tree was tested against 100 random trees to determine if it provided a significantly better fit to the sequence data than a random tree; this step was implemented to determine if phylogenetic signal could be detected in a given alignment. Random fixed topologies were generated with the rtree function from the ape R package, which randomly splits the edges; the evolutionary model from jModelTest for a particular gene fragment was used to re-estimate branch lengths with maximum likelihood in PhyML. Site-specific likelihoods were obtained using every random and maximum likelihood tree topology derived from the alignment using the --print_site_lnl option in PhyML. The obtained distributions of site-specific likelihoods were compared across trees using the Shimodaira -Hasegawa (SH) test [100] implemented in CONSELL v0.20 [101]. Only estimated gene trees providing much stronger support for sequence data than all 100 trees of random topology (SH-test *p*-value = 1) were included in further analyses. Although some gene fragments were short, phylogenetic signal still sufficed to obtain gene trees that better fit the sequence data than did random trees (Additional file 5: Table S3).

### Inferring species trees

The resulting gene trees were used to reconstruct species trees for every dataset (Fig. 1, step 4). Inference was carried out using all the gene trees that passed the SH test in Astral.4.7.12 [102] using the default parameter settings. Maximum likelihood gene trees were used to estimate the species tree topology. We also inferred 100 species trees using bootstrap replicates obtained during gene tree reconstruction and utilized them to evaluate clade support of the species tree.

### Obtaining a list of candidate hybridization events using clusters in gene trees

Gene trees were used to produce a list of candidate hybridization events by summarizing the topology of multilabeled gene trees obtained for every fragment in the form of a consensus network (Fig. 1, step 5). An online tutorial illustrating the procedures in this section and the section below has also been developed [103].

Although methods for inferring consensus networks have been implemented in a number of software packages, they were not applicable here due to computational burden on a dataset of the size we analyzed [104] or restrictions on input data (Dendroscope, for instance, does not allow multilabeled trees) [105]. We therefore developed a new procedure to obtain consensus networks on >20 species, represented by multiple individuals, from hundreds of gene trees.

Our method proceeds as follows. Clusters corresponding to clades observed in a gene tree (for every given clade, defined as a set of unique labels marking the leaves of the clades), and associated with sufficiently high bootstrap values, were collected along with the number of gene trees in which they were observed. Clusters observed in at least a certain percent of the gene trees were collected, irrespective of their compatibility. Thus, incompatible clusters included could potentially generate reticulation structures in the species history. For instance, clusters {A, B} and {B, C} cannot be generated by a species history that does not contain a reticulation event leading to {B}. Threshold percent values of 15, 20, 30, 40, and 50% were tested. After no more highly supported clusters were left, additional clusters were included if they were compatible with those already collected and therefore would not generate new reticulation events in the species tree. For instance, cluster {A, B} would not be included if cluster {B, C} is already in the set as then an additional reticulation, the one that leads to {B}, would be required. This was done in a greedy consensus fashion, more frequently observed clusters were considered first, and clusters observed in <2% of the gene trees were ignored. The final collection of clusters was summarized as a network, using a network-popping algorithm [106, 107]. Pseudo code appears in Additional file 4: Figure S6 and an implementation is available in an R script (Additional file 6). Networks were visualized using Dendroscope 3.2.8 [105] and appear in Additional file 4: Figure S7.

### Testing for ILS in putative hybridization events

Hybridization events detected in the cluster analysis at the consensus level of 15% or higher were further tested in an ILS-informed framework if they met two specific criteria. The level of 15% was used as the lowest threshold because the networks even at this level of confidence include a large number of hybridizations; a lower and more liberal threshold would be expected to yield less and less reliable candidates. The first criterion was that we only considered putative hybridization events involving diploid progenitors*.* Second, for polyploid species, we only tested hybridizations detected in datasets containing one polyploid at a time with polyploid species represented by haplotypes (datasets 6–14), as these datasets are expected to have more completely resolved gene trees than datasets containing additional polyploids. We also tested four cases of homoploid hybrid speciation detected in dataset 2 (diploids only, all consensus), and we tested the hybrid origin of *F. iturupensis* that was unsupported by the consensus network analysis, but that has been previously hypothesized (reviewed in [79, 108]).

In this step (Fig. 1, step 6), gene trees were utilized to test each putative hybridization event in an ILS-aware framework in STEM 2.0 [74] and PhyloNet [78]. For STEMhy analysis, gene trees were processed to retain only those tips corresponding to the potential hybrid and the possible progenitors as well as the outgroup lineage. The parameter θ, used by STEM 2.0 to convert gene tree branch lengths into coalescent units, was estimated locus-wise as the average distance between sequences from the same species, averaged across species included in each test. Multiple approaches for computing distances between sequences exist [109–112]; here for each sequence pair, distance was computed as a sum of branch lengths from the most recent common ancestor to the tips of the gene tree. The species tree was first inferred for a given collection of four species: the potential hybrid, two putative parental lineages, and the outgroup species (option 1 in STEM). Next, each potential hybridization event was tested in STEMhy using the same collection of gene genealogies, but supplying the inferred species tree estimated in the previous step and explicitly identifying the potential hybrid (option 3). Three alternative models, one involving hybridization and two without hybridization, were compared using the Akaike information criterion (AIC) reported by STEMhy. The model with the smallest AIC value was selected as best fitting the data (Table 1). To evaluate the robustness of the inference, hybridization events were tested using 100 bootstrap samples from the original set of loci, and STEMhy was applied in the same way as it was carried out for the original set of loci.

**Table 1:** STEMhy tests of putative hybridizations

Hybrid (ploidy)	Parents (ploidy)	Outgroup (ploidy of 2 is not shown)	Dataset	Level of Support	Δ AIC	Selected model (% from all bootstrap runs)	Estimated mixture fractions [95% bootstrap interval]
*F. chinensis* (2)	*F. pentaphylla* (2) *F. nipponica* (2)	*Drymocallis*	2	20%	302	hybr (88)	*F. pentaphylla:* 98 [2, 100] *F. nipponica:* 2 [0, 98]
*F. pentaphylla* (2)	*F. chinensis* (2) *F. nubicola* (2)	*Drymocallis*	2	20%	220	hybr (100)	*F. chinensis:* 8 [4, 79] *F. nubicola:* 92 [21, 96]
*F. daltoniana* (2)	*F. nilgerrensis* (2) *F. nipponica* (2)	*Drymocallis*	2	20%	Run failed		
*F. vesca* (2)	*F. mandshurica* (2) *F. bucharica* (2)	*Drymocallis*	2	40%	4317	hybr (100)	*F. mandshurica:* 97 [98, 93] *F. bucharica:* 3 [2, 7]
*F. corymbosa* (4)	*F. pentaphylla* (2) *F. chinensis* (2)	*Drymocallis*	6	15%	*F. corymbosa* was sister to a *F. pentaphylla/F. chinensis* clade in species tree inferred by STEM		
*F. gracilis* (4)	*F. pentaphylla* (2) *F. chinensis* (2)	*Drymocallis*	7	15%	*F. gracilis* was sister to a *F. pentaphylla*/*F. chinensis* clade in species tree inferred by STEM		
*F. moupinensis* (4)	*F. pentaphylla* (2) *F. chinensis* (2)	*Drymocallis*	8	20%	*F. moupinensis* was sister to a *F. pentaphylla*/*F. chinensis* clade in species tree inferred by STEM		
*F. orientalis* (4)	*F. mandshurica* (2) *F. vesca* (2)	*Drymocallis*	9	15%	*F. orientalis* was sister to a *F. mandshurica*/*F. vesca* clade in species tree inferred by STEM		
*F. tibetica* (4)	*F. pentaphylla* (2) *F. nubicola* (2)	*Drymocallis*	10	15%	9	no-hybr (63)	*F. pentaphylla: 100* [4, 100] *F. nubicola:* 0 [0, 96]
*F. moschata* (6)	*F. viridis* (2) *F. mandshurica* (2)	*Drymocallis*	11	20%	1	no-hybr (84)	*F. viridis:* 0 [0, 100] *F. mandshurica:* 100 [0, 100]
*F. moschata* (6)	*F. viridis* (2) *F. vesca* (2)	*Drymocallis*	11	20%	12,450	hybr (99)	*F. viridis:* 79 [72, 84] *F. vesca:* 21 [16, 28]
*F. moschata* (6)	*F. mandshurica* (2) *F. vesca* (2)	*Drymocallis*	11	30%	1	no-hybr (84)	*F. mandshurica:* 100 [0, 100] *F. vesca:*0 [0,100]
*F. chiloensis* (8)	*F. vesca* (2) *F. iinumae* (2)	*Drymocallis*	12	20%	21,892	hybr (100)	*F. vesca:* 9 [6, 34] *F. iinumae:* 91 [66, 94]
*F. virginiana* (8)	*F. vesca* (2) *F. iinumae* (2)	*Drymocallis*	13	20%	38,048	hybr (94)	*F. vesca:* 39 [32, 100] *F. iinumae:*61 [0, 68]
*F. iturupensis* (10)	*F. vesca* (2) *F. iinumae* (2)	*Drymocallis*	14	<15%	5996	hybr (67)	*F. vesca:*17 [0, 99] *F. iinumae* 83 [1, 100]

To conduct a comparable analysis in PhyloNet, gene genealogies were prepared in a similar way by trimming the gene trees to retain only those tips corresponding to the potential hybrid, possible progenitors, and the outgroup lineage. Because likelihood calculation in PhyloNet is computationally intensive, particularly with extensive intraspecific sampling, gene genealogies were trimmed to retain only up to 5 alleles per species, selected at random. To test each hybridization, a likelihood was computed for three scenarios of species evolution (one with hybridization and two without hybridization) using the CalGTProb function in PhyloNet. We supplied to PhyloNet the gene trees, the scenario of species evolution (represented by a network or tree), and a mapping of tips of gene trees to species. Parameter settings were: gene tree bootstrap threshold value, 15 for dataset 1 and 10 for all other datasets (−b 15 or -b 10); only topologies of the gene trees were used in the inference, not the branch lengths; and edge lengths and mixture fractions associated with hybridization events were estimated by the program (−o). AIC was used to compare models, as calculated using the log-likelihood produced by PhyloNet. The number of parameters was calculated as the number of species network (or tree) branches with estimated branch lengths plus one added parameter for each hybridization, as species contributions toward hybridization are also estimated by the program. The smallest-AIC model was selected; the AIC difference between the two best models is also reported (Table 2).

**Table 2:** PhyloNet tests of putative hybridizations

Hybrid (ploidy)	Parents (ploidy)	Outgroup (ploidy = 2)	Dataset	Level of Support	Δ AIC	Selected model	Estimated mixture fractions
*F. chinensis* (2)	*F. pentaphylla* (2) *F. nipponica* (2)	*Drymocallis*	2	20%	10	hybr	*F. pentaphylla:* 10 *F. nipponica:* 90
*F. pentaphylla* (2)	*F. chinensis* (2) *F. nubicola* (2)	*Drymocallis*	2	20%	8	no-hybr	*F. chinensis:* 0 *F. nubicola:* 100
*F. daltoniana* (2)	*F. nilgerrensis* (2) *F. nipponica* (2)	*Drymocallis*	2	20%	20	hybr	*F. nilgerrensis:* 64 *F. nipponica:* 36
*F. vesca* (2)	*F. mandshurica* (2) *F. bucharica* (2)	*Drymocallis*	2	40%	126	hybr	*F. mandshurica:* 72 *F. bucharica:* 28
*F. corymbosa* (4)	*F. pentaphylla* (2) *F. chinensis* (2)	*Drymocallis*	6	15%	57	hybr	*F. pentaphylla:* 45 *F. chinensis:* 55
*F. gracilis* (4)	*F. pentaphylla* (2) *F. chinensis* (2)	*Drymocallis*	7	15%	44	hybr	*F. pentaphylla:* 62 *F. chinensis:* 38
*F. moupinensis* (4)	*F. pentaphylla* (2) *F. chinensis* (2)	*Drymocallis*	8	20%	33	hybr	*F. pentaphylla:* 68 *F. chinensis:* 32
*F. orientalis* (4)	*F. mandshurica* (2) *F. vesca* (2)	*Drymocallis*	9	15%	56	hybr	*F. mandshurica:* 79 *F. vesca:* 21
*F. tibetica* (4)	*F. pentaphylla* (2) *F. nubicola* (2)	*Drymocallis*	10	15%	24	hybr	*F. pentaphylla:* 52 *F. nubicola:* 48
*F. moschata* (6)	*F. viridis* (2) *F. mandshurica* (2)	*Drymocallis*	11	20%	151	hybr	*F. viridis:* 8 *F. mandshurica:* 92
*F. moschata* (6)	*F. viridis* (2) *F. vesca* (2)	*Drymocallis*	11	20%	340	hybr	*F. viridis:* 59 *F. vesca:* 41
*F. moschata* (6)	*F. mandshurica* (2) *F. vesca* (2)	*Drymocallis*	11	30%	66	hybr	*F. mandshurica:* 90 *F. vesca:* 10
*F. chiloensis* (8)	*F. vesca* (2) *F. iinumae* (2)	*Drymocallis*	12	20%	511	hybr	*F. vesca:* 18 *F. iinumae:* 82
*F. virginiana* (8)	*F. vesca* (2) *F. iinumae* (2)	*Drymocallis*	13	20%	408	hybr	*F. vesca:* 10 *F. iinumae:* 90
*F. iturupensis* (10)	*F. vesca* (2) *F. iinumae* (2)	*Drymocallis*	14	<15%	285	hybr	*F. vesca:*3 *F. iinumae* 97

## Results

### Targeted capture sequencing and assembly

A total of 1419 exons and adjacent intron and untranscribed regions from 257 nuclear loci were targeted for sequencing in 53 individuals from 20 *Fragaria* species with ploidy number ranging from 2 to 10 and one individual from a diploid outgroup, *Drymocallis glandulosa*. From the 54 samples, 725,442,456 total reads were obtained after trimming (856 to 167,300,000 per individual) from two lanes of 101-bp single-end reads (170,386,591 reads) and two lanes of 101-bp paired-end reads (543,074,857 reads) on the Illumina HiSeq 2000, and one lane of 250-bp paired-end reads (11,981,008 reads) on the Illumina MiSeq. Of these reads, 48.35% aligned to the reference (19.44 to 78.61% across individuals), generating an average depth of 52 reads per position (4 to 387). The 39 *Fragaria* individuals with sufficient coverage (see Methods, Sequence and Haplotype Assembly, point 6) were included in further analyses (Fig. 1, Additional file 1: Table S1). The total length of the obtained consensus assembly was 33,797,493 bp (576,013 to 1,136,434 bp across individuals).

### Sequence variability

Reference-based assembly, variant-calling, and variant analysis identified 183,253 total variable sites in the 40 individuals. The frequency of variable sites differed across genes and individuals (Additional file 4: Figure S8), ranging from 0 (in 21.5% of genes across individuals) to 2.3% (in gene30425, encoding probable polyol transporter 4, in a single *F. bucharica* individual, ID CFRA522, index 39). On average, *F. iturupensis* had the highest frequency of heterozygous positions averaged across individuals and genes (0.28%), and *F. daltoniana* had the lowest (0.014%), with an overall average of 0.14% across all of the individuals and genes.

Phylogenetic distances were calculated between consensus sequences for all interspecific comparisons (Additional file 4: Figure S9). Average interspecific distances showed a five-fold difference, ranging from 0.006 (*F. orientalis* to *F. mandshurica*) to 0.033 (*F. tibetica* to *F. iturupensis*) when averaged across loci and individuals.

### Haplotype assembly and analysis

Genotypes were phased using linkage information in Illumina reads to reconstruct haplotypes for each of the 257 genes for every individual. Phasing success varied across genes and individuals and between diploids and polyploids (Additional file 4: Figure S10). The average length of the longest haplotype across all individuals and all loci was 3387 bp (Additional file 4: Figure S10). In total, 62% of heterozygous positions were incorporated into the longest haplotype. In 28% of genes (diploids, 23%; polyploids, 37%), all heterozygous positions were phased in the largest haplotype, representing the full length of the gene (Additional file 4: Figure S10).

Pairwise distances between haplotypes from the same *Fragaria* species ranged from 6.9 × 10^−4^ for *F. daltoniana* to 1.07 × 10^−2^ for *F. iturupensis*, averaging 3.94 × 10^−3^ (Additional file 4: Figure S3). Polyploids had larger average within-species pairwise distances between haplotypes than did diploids (5.3 × 10^−3^ compared to 2.8 × 10^−3^). *Fragaria chinensis* and *F. viridis* possessed the largest intraspecies distances among diploid species, 6.24 × 10^−3^ and 5.37 × 10^−3^, respectively (Additional file 4: Figure S3). These two diploids also had the highest frequency of heterozygous positions among all included taxa (Additional file 4: Figure S8). The three taxa with the highest ploidy, *F. virginiana* (8×)*, F. chiloensis* (8×)*,* and *F. iturupensis* (10×), had the most within-species variability (9.1 × 10^−3^, 9.4 × 10^−3^ and 1.07 × 10^−2^, respectively; Additional file 4: Figure S3).

### Species phylogenies

**Fig. 3:**
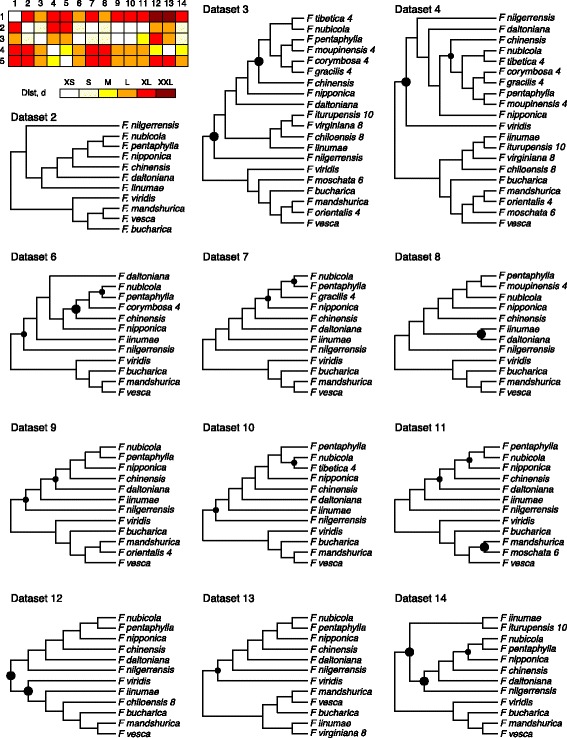
Species trees reconstructed using ASTRAL for various sets of taxa. Topologies were tested using bootstrap trees generated by PhyML. Clades observed in >85% of bootstrap replicates are not labeled, clades observed in 70 to 85% of replicates are marked with small circles, and those observed in <70% of replicates are marked with large circles. Ploidy levels are shown for non-diploid species. Branch lengths are not to scale. Species trees for datasets 1 and 5 are not shown, but they are included in the supplementary material (Additional file 4: Figure S11). Distances between topologies of reconstructed species trees are calculated as described in the text, and they are plotted in the heat map at the top left. Distance ranges are encoded as follows: XS: [0, 0.1], S: (0.1, 0.2], M: (0.2, 0.3], L: (0.3, 0.4], XL: (0.4, 0.5], XXL: (0.5, 0.6]

We used the trees for each of the gene fragments to estimate relationships among *Fragaria* species in every dataset (Fig. 2), employing a coalescence-based analysis implemented in ASTRAL (Fig. 3, Additional file 4: Figure S11). Species-tree reconstruction for full and partial datasets are generally concordant, with the exception of datasets 1 and 5, for which haplotypes were used for all species (diploid taxa only or all taxa, respectively). These two phylogenies largely disagree with each other and with phylogenies inferred from other datasets (see heat map in Fig. 3), and they include clades unsupported by geography and morphology. As an example of incongruence involving these two datasets, *F. daltoniana* and *F. iinumae* are recovered as a clade sister to the remaining species in dataset 1, whereas dataset 5 shows a relationship between these two species commonly reflected in the remaining datasets (Fig. 1, Additional file 4: Figure S11). In contrast, both datasets 1 and 5 place *F. nilgerrensis* as sister to the “vesca” clade (Additional file 4: Figure S11), whereas other datasets generally place *F. nilgerrensis* as related to the “China” clade (Fig. 3). This difference among datasets is further highlighted by low bootstrap support (<70%) for a number of clades in the species trees inferred for datasets 1 and 5. The inconsistency between datasets 1 and 5 and the other datasets is likely attributable to insufficient phylogenetic signal to resolve gene trees when including a large number of individuals as haplotypes. In comparing datasets 1 and 2, the sums of total sites and variable sites are (85,485; 7328) and (454,409; 39,068), respectively, and they are (281,984; 36,390) and (40,736; 5116) for datasets 4 and 5. For these reasons, all further analyses focus on the datasets in which the diploid species are represented by consensus sequences (datasets 2 to 4 and 6 to 14).

Species trees from datasets 2 (diploids only, all consensus sequence) and 3 (all taxa, all consensus sequence) are similar for the diploid species. Inclusion of the polypoid species (dataset 3) supports the presence of a China clade. However, the canonical vesca clade is split, with the octoploid and decaploid species more closely related to *F. iinumae* instead of *F. vesca*, likely reflecting the high contribution of *F. iinumae* to the nuclear genome of these species. Species trees based on datasets 3 and 4 (all taxa, haplotypes for polyploids) contain many of the same well-supported clades, but they differ broadly in the basal topology of the tree and the phylogenetic position of both *F. viridis* and *F. nilgerrensis* (Fig. 3).

For polyploid ancestry, both *F. corymbosa* (4×) and *F. gracilis* (4×) are sister to a *F. nubicola* (2×) and *F. pentaphylla* (2×) clade when they are included individually (datasets 6 and 7, respectively), but they form a sister relationship to a clade that includes *F. pentaphylla* and *F. moupinensis* (but not *F. nubicola*) when both species are present (datasets 3 and 4). This result is consistent with *F. pentaphylla* being a progenitor of these two tetraploid species, and is suggestive that *F. nubicola* might be the other progenitor.

*Fragaria moupinensis* (4×) consistently emerges as sister to *F. pentaphylla* (datasets 3, 4, and 8), and *F. tibetica* (4×) forms a clade with *F. nubicola* (datasets 3, 4, and 10). This result suggests that *F. pentaphylla* and *F. nubicola* are likely to be diploid progenitors of tetraploids *F. moupinensis* and *F. tibetica*, respectively. *Fragaria orientalis* (4×) and *F. moschata* (6×) often comprise a well-supported clade with the diploid *F. mandshurica* (datasets 3, 4, 9, and 11), though *F. vesca* is also closely allied with these polyploids (datasets 4, 9, and 11 in particular).

If the two octoploids and one decaploid are included, they consistently form a clade with the diploid *F. iinumae* (datasets 3, 4). When analyzed individually, *F. virginiana* (8×) and *F. iturupensis* (10×) are resolved as sister to *F. iinumae* (datasets 13 and 14, respectively). In contrast, the position of *F. chiloensis* (8×) is more strongly influenced by the contribution to its ancestry from the *F. bucharica, F. vesca,* and *F. mandshurica* clade (dataset 12).

### Hybridization events detected using a consensus approach

**Fig. 4:**
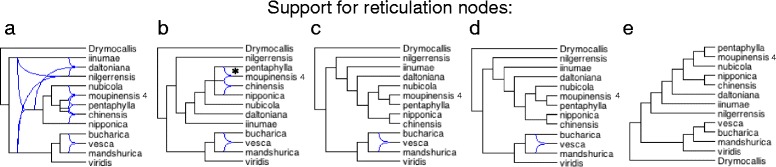
Cluster networks for dataset 8, constructed using all fragments passing the SH test against 100 random trees. The percent indicates the minimum support required for a cluster to be included in the procedure for identifying putative hybridizations. (**a**) 15. (**b**) 20. (**c**) 30. (**d**) 40. (**e**) 50%. The asterisk indicates a potential hybridization event leading to formation of tetraploid *F. moupinensis* at the highest confidence level. Networks for all datasets appear in Additional file 4: Figure S7

Species tree reconstruction with ASTRAL produced topologies and major clades that are mostly consistent with previous reports on *Fragaria* evolution. However, species tree approaches do not seek to detect the hybridizations that might have led to the emergence of allopolyploid species. For this reason, consensus network analysis was performed to identify a list of putative hybridization events. Setting thresholds for the consensus networks at 15, 20, 30, 40 or 50%, robust patterns were detected that were consistent with hybridizations in gene tree topologies for a majority of the datasets (Fig. 4 for dataset 8, Additional file 4: Figure S7 for all datasets).

To identify promising hybridizations, we evaluated consensus networks for each dataset, starting with conservative thresholds (50 and 40%) and moving to more sensitive values (30, 20, and 15%) until a potential hybridization event yielding a polyploid was detected. For instance, in dataset 8 (Fig. 4), such a potential hybridization is detected at a level of 20%.

In dataset 4 (all taxa, haplotypes for polyploids), all polyploid species except for *F. gracilis* show signs of hybrid ancestry in the 20% consensus tree. For *F. corymbosa*, the parentage appears to be a cross between *F. gracilis* and *F. chinensis*. The ancestry of the remaining polyploids appears more complex. *Fragaria moupinensis* is descended from a mixture of *F. pentaphylla*, *F. nubicola,* and the tetraploid *F. tibetica*. *Fragaria tibetica* itself is derived from a mixture of *F. nubicola, F. pentaphylla,* and the tetraploid *F. moupinensis. Fragaria orientalis* appears to derive from a mixture of *F. mandshurica* and *F. viridis,* and the hexaploid *F. moschata. Fragaria moschata* appears to be derived from a mixture of the diploids *F. viridis* and *F. mandshurica* and the tetraploid *F. orientalis.* The only diploid indicated in the parentage of the highly polyploid species *F. chiloensis, F. virginiana*, and *F. iturupensis* is *F. iinumae*.

Three polyploid species among nine analyzed show evidence of hybrid ancestry in datasets that included one polyploid at a time (Additional file 4: Figure S7, datasets 6 to 14) in the 20% consensus network (*F. moupinensis, F. virginiana,* and *F. chiloensis*). *Fragaria moschata* has three potential sets of diploid progenitors, and these hybridizations are supported in the 20 or 30% consensus networks. Four additional polyploids (*F. corymbosa, F. gracilis, F. orientalis,* and *F. tibetica*) show evidence of hybrid ancestry in the 15% consensus networks (Additional file 4: Figure S7). The decaploid *F. iturupensis* is consistently sister to *F. iinumae,* and it possesses no hybrid ancestry at any level of support for the reticulation nodes (Additional file 4: Figure S7).

Among the China clade tetraploids, *F. moupinensis* is suspected to be derived from the hybridization of *F. pentaphylla* and *F. chinensis* (Additional file 4: Figure S7, dataset 8, 20% threshold), perhaps with a contribution from *F. nipponica*, although *F. pentaphylla* is also highlighted as a potential progenitor in dataset 4. Both *F. corymbosa* and *F. gracilis* also appear to be hybrids of *F. pentaphylla* and *F. chinensis* (Additional file 4: Figure S7, datasets 6 and 7, 15% threshold), although the network is more complex and has reduced support compared to that for *F. moupinensis*. The contribution from *F. chinensis* is also detected in dataset 4 for *F. corymbosa*, but the contribution from *F. pentaphylla* is not. *Fragaria tibetica* is hypothesized to be descended from *F. nubicola* in both the phylogeny with complete taxonomic sampling (Additional file 4: Figure S7, dataset 4, 20% network) and the one with *F. tibetica* as the sole polyploid (Additional file 4: Figure S7, dataset 10, 15% network). However, differences in additional potential contributors are apparent between those two datasets.

*F. orientalis* appears to be derived from hybridization between *F. mandshurica* and *F. vesca* (Additional file 4: Figure S7, dataset 9, 15% network), with a potential contribution from *F. bucharica*. Dataset 4 corroborates the *F. mandshurica* contribution, although it identifies *F. viridis* and *F. moschata* as contributing lineages. Ancestry of the hexaploid *F. moschata* appears to trace to three diploid species: *F. viridis, F. mandshurica,* and *F. vesca,* but the ancestry signals from *F. mandshurica* and *F. vesca* are more robust and detectable even at 30% support for reticulation nodes (Additional file 4: Figure S7, dataset 11). *F. bucharica* might also be a potential ancestor, as gene exchange in this clade is supported at the 30% level. Support for the contribution of *F. viridis* and *F. mandshurica* is seen in dataset 4, which also suggests *F. orientalis* as a progenitor of *F. moschata* (Additional file 4: Figure S7, dataset 4, 20% network).

The octoploids *F. chiloensis* and *F. virginiana* appear to be derived from hybridization between *F. iinumae* and a combination of *F. vesca, F. mandshurica,* and *F. bucharica* (Additional file 4: Figure S7, datasets 12 and 13), with support found at the 30% level for *F. chiloensis* and at 20% for *F. virginiana*. When considering only diploids, consensus detects no evidence of hybrid ancestry of the decaploid *F. iturupensis*, even at the lowest threshold. Only *F. iinumae* is detected as a sister clade in this dataset (Additional file 4: Figure S7, dataset 14).

Suggested ancestral lineages for polyploids are largely consistent between dataset 4, which includes all polyploids, and datasets 6–14, which include one polyploid species each. However, the differences are not unexpected due to different taxon sampling and different gene tree resolution in different datasets. Some of the potential progenitors identified are also consistent with previous reports, whereas others have not previously been suggested.

### Determining hybridization versus ILS through model testing

Extensive ILS associated with short species tree branches might lead many gene trees to deviate from a species tree topology, generating gene tree discordance patterns similar to the one expected under hybridization. Therefore, gene tree discordance alone might not be an accurate indication of hybridization, and hybridizations detected using a consensus species network approach might be “false positives” due to ILS. For this reason, some putative hybridizations (see Methods section) identified with the consensus approach were evaluated in hypothesis-testing frameworks using PhyloNet and STEMhy, which allow detection of hybridization in the presence of ILS using gene tree topologies, with or without branch lengths (Tables 1 and 2).

Among the diploids, four putative cases of hybridization were tested with STEMhy and PhyloNet, and two of these cases were confirmed by both methods (*F. chinensis* and *F. vesca*; Tables 1 and 2). Both analyses confirm gene flow between *F. vesca* and *F. bucharica* and *F. mandshurica*. According to STEMhy, 97% of the *F. vesca* genome is descended from *F. mandshurica* and 3% from *F. bucharica,* whereas PhyloNet suggests corresponding values of 72 and 28%, respectively. This result highlights limited evidence for traditional species delimitation among these 3 taxa, and potentially suggests that the lineages should be considered a single species. *Fragaria chinensis* is supported as having hybrid origin by both methods, but the estimates of ancestral contributions are dissimilar (Tables 1 and 2). STEMhy has the ratio of contributions of *F. pentaphylla* to *F. nipponica* as 98:2 whereas PhyloNet reports 10:90.

According to PhyloNet, *F. daltoniana* also records gene flow in its history, with 64% of its genome descended from *F. nilgerrensis* and 36% from *F. nipponica*. However, as this finding is not confirmed by STEMhy and is not supported by morphology of the species or current geographic distribution, it should be viewed with caution. According to STEMhy, 92% of the *F. pentaphylla* genome is inferred to have *F. nubicola* origin, with 8% coming from *F. chinensis*; however, PhyloNet does not detect this gene flow. We note that an absence of haplotype sequences for diploids has made it difficult to confidently assess homoploid hybridizations.

For polyploids, STEMhy and PhyloNet were used to test 11 hybridization scenarios involving 9 potential hybrid species (Tables 1 and 2). Hybridization is confirmed in 4 by both programs (*F. moschata, F. chiloensis, F. virginiana, F. iturupensis*), whereas the remaining 7 are confirmed only by PhyloNet. In four of the cases (Table 1), STEMhy resolved the polyploid as sister to the putative diploid progenitors confirmed by PhyloNet (Table 2). This behavior of STEMhy might be attributed to the fact that it relies on gene tree branch length, which is hard to estimate in empirical studies. Three of 11 hybridization scenarios are alternative hypotheses for the origin of *F. moschata,* with only one of these hypotheses (*F. viridis* plus *F. vesca*) supported by both methods. In this latter case, the estimated *F. viridis* proportion (59% Phylonet, 79% STEMhy) suggests that two subgenomes originated with this species, and one from *F. vesca.* Although a hexaploid can have three different diploid ancestors, when one of the three ancestors is omitted, it seems logical to expect a ratio between 1:2 and 2:1 for the genomic contributions of the other two ancestors, depending on the phylogenetic distance between the missing ancestor and the ones included, rather than the skewed ratios (9:1) estimated by PhyloNet for the hypotheses about *F. moschata* origins rejected by STEMhy. The octoploids *F. chiloensis* and *F. virginiana* and the decaploid *F. iturupensis* are all supported as originating from hybridizations of *F. vesca* and *F. iinumae*, with mixture fractions suggesting a larger contribution from *F. iinumae* in all polyploids across both methods (Tables 1 and 2).

## Discussion

### Strategy employed for testing hybrid origins of species

A number of methods have been proposed for using multiple gene trees to infer species networks, which incorporate hybridization events important in the evolution of many taxa [34, 77, 88, 113–115]. However, these methods are often challenging to apply, either because of computational overhead for ILS-aware likelihood-based methods or due to potentially misleading results for consensus methods naïve with regard to the coalescent process. We therefore designed a “hybrid” strategy that uses consensus to propose hybridizations in a species history and likelihood to test putative hybridizations in an ILS-aware framework (Fig. 1). This strategy avoids the computational burden of inferring species networks by de-novo likelihood construction, but it also evaluates and potentially excludes hybridizations erroneously identified by consensus approaches due to pervasive ILS along short branches in a species’ history.

We designed this strategy to test potential hybrid origins of polyploid species in the genus *Fragaria*. Although we sought to identify hybridizations associated with allopolyploidy, our protocol can also be applied to hybridizations associated with homoploid evolution (hybrid speciation at the diploid level). It can potentially be applied to infer entire species networks by testing every putative hybridization, adding reticulation to a “background” species tree. A similar approach has been proposed for inferring reticulate species histories from SNP data [116] and gene genealogies [117], and it has been shown to perform well in simulations [117].

Interestingly, 14 of 15 candidate *Fragaria* hybridizations that were identified using consensus were also supported by ILS-informed inference using PhyloNet (Table 2). By contrast, only 7 of 15 successfully tested candidate hybridizations were supported by STEMhy (one failed run; Table 1). Most polyploid *Fragaria* are suspected to be allopolyploids [79], and therefore, evidence of hybrid origin of these species is expected. While PhyloNet confirmed 15 of 16 tested scenarios, and we suggest caution in interpreting these results, it should be noted that each of these hybridization events was supported in many individual gene trees, with significant support in the cluster networks (Fig. 4 and Additional file 4: Figure S7). The discrepancy in results between STEMhy and PhyloNet likely arises from the fact that likelihood-based methods are sensitive to errors known to be pervasive in empirical gene trees, even at the level of gene tree topologies [117]. Because STEMhy and PhyloNet differ in their response to gene tree error and are prone to different types of errors themselves [117], we suggest that increasing confidence should be placed on those candidate hybridization events supported by both methods (6 events in Tables 1 and 2).

### Haplotype assembly for phylogeny of polyploids

Individual haplotype sequences were inferred using linkage information present in Illumina reads. This strategy is rarely applied for phylogenetics and is underused in polyploids in comparison with consensus assemblies [43, 44, 118]. For scenarios with di- or heterosomic inheritance, in which alleles from homeologous chromosomes are unable to coalesce prior to the ancestral lineage in which they co-existed as homologs, combining homeologous alleles into a consensus eliminates a signal of allopolyploid ancestry in individual gene trees. The consequences of such a data-processing strategy for gene tree topologies and for species network inferences from those topologies are unknown, though they can be assessed by simulation.

A challenge for our alternative approach is that haplotype assembly remains a formidable task, and loss of information due to short lengths of contiguous haplotype assembly can lead to poorly resolved or erroneous gene trees. For example, among the polyploid *Fragaria*, averaged over 257 genes and all species, haplotype assembly blocks averaged 80% of the complete target (ranging from 53 to 100%), leaving many assemblies with too little data to confidently estimate gene trees. This concern is largely circumvented using consensus assemblies, as assembled regions can simply be concatenated to achieve better resolution of gene trees. However, as mentioned above, concatenation comes with the loss of resolved haplotypes.

Haplotype assembly success varied across genes, individuals, and species. In particular, polyploids yielded more complete assemblies than diploids (Additional file 4: Figure S10). Averaged over all loci and all species, the longest haplotype assembly blocks for diploids contained only 37% of all heterozygous positions, whereas the longest haplotype per gene in polyploids contained 73.5% of heterozygous sites. This pattern differs from previous reports, in which haplotype assembly success was correlated negatively with ploidy [55, 56]. We offer two possible explanations. First, given the greater sequence divergence between homeologous sequences in polyploids, the increased variation likely aids in linkage of the short-read sequence data. Second, polyploid individuals were sequenced on the Illumina MiSeq with 250-bp paired-end reads, whereas diploids were sequenced by Illumina HiSeq with 101-bp reads (a combination of single-end and paired-end reads). Again, longer reads provide a greater opportunity to link variable sites separated by long stretches of homozygosity [119]. Hence, we recommend using the longest reads available on a sequencing platform and utilizing paired-end reads as available [87, 120]. Additionally, using longer inserts or variable insert size in sequencing could also help in linking variable positions together, as has previously been observed [121].

Several recent studies on polyploid evolution have used haplotype sequences from nuclear genes to reconstruct species networks [41, 113]. Due to the techniques employed, in which alleles are physically isolated prior to sequencing, these studies have utilized few genes; the use of a small number of loci in species tree reconstruction or network inference can lead to erroneous species tree estimates [77, 114, 117]. Target capture and sequencing followed by haplotype assembly can address this concern by obtaining haplotype information for a large number of nuclear markers (257 nuclear genes). The approach both increases the accuracy of species network construction and has the potential to accurately identify a greater number of reticulation events associated with hybridization compared to use of fewer genes.

### Hybrid origins of polyploid *Fragaria* species

This study is the first phylogenetic study to include all currently recognized species in the China clade. Previous studies using one [79, 81] or two [80] species did not resolve hybrid origins of the tetraploids of this clade. Our PhyloNet analyses supported involvement of the diploid *F. pentaphylla* in the ancestry of tetraploids *F. corymbosa, F. gracilis, F. moupinensis*, and *F. tibetica* (Table 2). *Fragaria chinensis* was supported as the other progenitor of the first three, whereas *F. nubicola* was supported for *F. tibetica.* In contrast, STEMhy resolved the four tetraploids as sister to these same diploid pairs, indicating potentially better performance of PhyloNet in resolving the China clade (Table 1). Morphological evidence has been used to propose *F. chinensis* as an ancestor of *F. gracilis* and *F. corymbosa* [78], consistent with PhyloNet and some consensus networks (Additional file 4: Figure S7). Based on morphological similarity, *F. tibetica* has been proposed as an autotetraploid derivative of *F. pentaphylla* [122]. The association of *F. nubicola* and *F. tibetica* in PhyloNet and ASTRAL (Fig. 3, dataset 4) and some consensus networks (Additional file 4: Figure S7) argues against this hypothesis.

The remaining tetraploid, *F. orientalis*, has long been associated with the vesca clade. Staudt [123] described the diploid *F. mandshurica* as a distinct species, and proposed that *F. orientalis* was its autotetraploid descendant. Here, ASTRAL recovered *F. mandshurica, F. vesca* and *F. moschata* as sisters of *F. orientalis*. PhyloNet also confirms *F. mandshurica* and *F. vesca* as progenitors of the tetraploid *F. orientalis.* The hexaploid *F. moschata* has been considered a hybrid of diploids *F. viridis* and *F. vesca* [80]; this claim is supported by both STEMhy and PhyloNet. Our results also indicate potential ancestry involving *F. mandshurica* (PhyloNet, but not STEMhy), which has not been previously proposed. This signal raises the possibility that *F. moschata* has one subgenome from each of these 3 species. Genetic linkage mapping will be required to substantiate this hypothesis.

Phylogenetic analysis of genetically mapped linkage groups has confirmed a single origin of the octoploids *F. chiloensis* and *F. virginiana*, and identified *F. vesca* subsp. *bracteata, F. iinumae,* and two genomes corresponding to an extinct ancestor closely related to *F. iinumae* as progenitors of the octoploids [30]. Our STEMhy and PhyloNet results show a greater contribution of *F. iinumae* than *F. vesca* to the ancestry of the octoploids, though most estimates exceed the predicted 3:1 mixture fraction. The dominant *F. iinumae* ancestry is also observed with ASTRAL and most of the consensus network results (Fig. 3 and Additional file 4: Figure S7).

The STEMhy analyses (Table 1) estimated 17% *F. vesca* ancestry in the decaploid *F. iturupensis*. This outcome could be attributable to recent hybridization between an octoploid *F. iturupensis* [112] and the diploid *F. iinumae*, resulting in four *F. iinumae* subgenomes and one *F. vesca* (expected 20%). Surprisingly, the Phylonet estimate is only 3% *F. vesca* ancestry in *F. iturupensis*. However, this is consistent with its underestimates of 10 and 18% *F. vesca* ancestry (vs. 25% expected) in the octoploids *F. virginiana* and *F. chiloensis*, respectively. The difficulty in estimating the ancestry of the octoploid and decaploid species demonstrates the challenge of deciphering higher-order polyploidy even with a large set of gene trees.

## Conclusions

In summary, here we designed a protocol to assess reticulate ancestry of diploid and polyploid *Fragaria* species using next-generation sequencing data. The approach makes use of individual-level sequences and haplotypes, employing multiple loci in a consensus framework to propose hybridizations, and testing those hybridizations using likelihood. We have suggested novel phylogenetic hypotheses that contribute to an understanding of genome evolution in *Fragaria*, at the same time demonstrating that for a clade with multiple ploidies represented, even a relatively large dataset can be insufficient to confidently resolve the entire hybridization history. A challenge in the analysis has been the complexity of the processing required to make the data from various species of different ploidy commensurable, and we have made a number of simplifications. We expect that as both NGS technology and computational techniques for processing reads in polyploids improve, it will be increasingly possible to produce high-quality haplotypes that can enable refined inferences of the evolutionary histories of polyploids.

## Additional files

Additional file 1: Table S1. List of biological samples. (XLSX 31 kb)

Additional file 2: Fasta sequences from the *F. vesca* genome used for reference-based assembly. (FA 1130 kb)

Additional file 3: Table S2. Description of genes targeted by probes. (TXT 31 kb)

Additional file 4: Supplementary materials. Figs. S1-S11. (PDF 3514 kb)

Additional file 5: Table S3. Gene trees for all of the datasets. (TXT 7807 kb)

Additional file 6: R code for constructing consensus networks from multilabeled gene trees. (R 6 kb)

## Abbreviations

AIC: Akaike information criterion
ILS: Incomplete lineage sorting
NGS: Next-generation sequencing
SH: Shimodaira-Hasegawa

## References

[1] Otto SP, Whitton J (2000). Polyploid incidence and evolution. Annu Rev Genet.

[2] Adams KL, Wendel JF (2005). Polyploidy and genome evolution in plants. Curr Opin Plant Biol.

[3] Madlung A (2013). Polyploidy and its effect on evolutionary success: old questions revisited with new tools. Heredity.

[4] Blomme T, Vandepoele K, De Bodt S, Simillion C, Maere S, Van de Peer Y (2006). The gain and loss of genes during 600 million years of vertebrate evolution. Genome Biol.

[5] Jiao Y, Wickett NJ, Ayyampalayam S, Chanderbali AS, Landherr L, Ralph PE (2011). Ancestral polyploidy in seed plants and angiosperms. Nature.

[6] Mallet J (2007). Hybrid speciation. Nature.

[7] Chester M, Gallagher JP, Symonds VV, Cruz da Silva AV, Mavrodiev EV, Leitch AR (2012). Extensive chromosomal variation in a recently formed natural allopolyploid species, *Tragopogon miscellus* (Asteraceae). Proc Natl Acad Sci U S A.

[8] Soltis DE, Mavrodiev EV, Meyers SC, Severns PM, Zhang L, Gitzendanner MA (2012). Additional origins of Ownbey’s *Tragopogon mirus*. Bot J Linn Soc.

[9] Arkhipova IR, Rodriguez F (2013). Genetic and epigenetic changes involving (retro) transposons in animal hybrids and polyploids. Cytogenet Genome Res.

[10] Vallejo-Marín M, Buggs RJA, Cooley AM, Puzey JR (2015). Speciation by genome duplication: repeated origins and genomic composition of the recently formed allopolyploid species *Mimulus peregrinus*. Evolution.

[11] Wood TE, Takebayashi N, Barker MS, Mayrose I, Greenspoon PB, Rieseberg LH. The frequency of polyploid speciation in vascular plants. Proc Natl Acad Sci U S A. 2009;106:13875–9. 10.1073/pnas.0811575106 PMC2728988 19667210

[12] Weiss-Schneeweiss H, Emadzade K, Jang T-S, Schneeweiss GM. Evolutionary consequences, constraints and potential of polyploidy in plants. Cytogenet Genome Res. 2013;140:137-50. 10.1159/000351727 PMC3859924 23796571

[13] Otto SP (2007). The evolutionary consequences of polyploidy. Cell.

[14] Mayrose I, Zhan SH, Rothfels CJ, Magnuson-Ford K, Barker MS, Rieseberg LH (2011). Recently formed polyploid plants diversify at lower rates. Science.

[15] Soltis DE, Segovia-Salcedo MC, Jordon-Thaden I, Majure L, Miles NM, Mavrodiev EV (2014). Are polyploids really evolutionary dead-ends (again)? A critical reappraisal of Mayrose *et al.* (2011). New Phytol.

[16] Fawcett JA, Maere S, Van de Peer Y. Plants with double genomes might have had a better chance to survive the Cretaceous–Tertiary extinction event. Proc Natl Acad Sci U S A. 2009;106:5737–42. 10.1073/pnas.0900906106 PMC2667025 19325131

[17] Vanneste K, Baele G, Maere S, Van de Peer Y. Analysis of 41 plant genomes supports a wave of successful genome duplications in association with the Cretaceous-Paleogene boundary. Genome Res. 2014;24:1334-47. 10.1101/gr.168997.113 PMC4120086 24835588

[18] Leitch AR, Leitch IJ (2008). Genomic plasticity and the diversity of polyploid plants. Science.

[19] Liu S, Liu Y, Yang X, Tong C, Edwards D, Parkin IAP, et al. The *Brassica oleracea* genome reveals the asymmetrical evolution of polyploid genomes. Nat Commun. 2014;5:3930. 10.1038/ncomms4930 PMC4279128 24852848

[20] Moghe GD, Shiu S-H (2014). The causes and molecular consequences of polyploidy in flowering plants. Ann N Y Acad Sci.

[21] Renny-Byfield S, Wendel JF (2014). Doubling down on genomes: polyploidy and crop plants. Am J Bot.

[22] Gregory TR, Mable BK. Polyploidy in animals. In: TR Gregory, ed. The Evolution of the Genome. Burlington, MA: Academic Press; 2011. pp. 427–517.

[23] Thompson JD, Lumaret R (1992). The evolutionary dynamics of polyploid plants: origins, establishment and persistence. Trends Ecol Evol.

[24] Ramsey J, Schemske DW (1998). Pathways, mechanisms, and rates of polyploid formation in flowering plants. Annu Rev Ecol Syst.

[25] Ramsey J, Schemske DW (2002). Neopolyploidy in flowering plants. Annu Rev Ecol Syst.

[26] Giraud T, Refrégier G, Le Gac M, de Vienne DM, Hood ME. Speciation in fungi. Fungal Genet Biol. 2008;45:791–802. 10.1016/j.fgb.2008.02.001 18346919

[27] Kim S-T, Sultan SE, Donoghue MJ. Allopolyploid speciation in *Persicaria* (Polygonaceae): insights from a low-copy nuclear region. Proc Natl Acad Sci U S A. 2008;105:12370–5. 10.1073/pnas.0805141105 PMC2517606 18711129

[28] Ainouche ML, Wendel JF. Polyploid speciation and genome evolution: lessons from recent allopolyploids. In: P Pontarotti, ed. Evolutionary Biology: Genome Evolution, Speciation, Coevolution and Origin of Life. New York City: Springer; 2014. pp. 87–113.

[29] Marcussen T, Sandve SR, Heier L, Spannagl M, Pfeifer M, International Wheat Genome Sequencing Consortium (2014). Ancient hybridizations among the ancestral genomes of bread wheat. Science.

[30] Tennessen JA, Govindarajulu R, Ashman T-L, Liston A (2014). Evolutionary origins and dynamics of octoploid strawberry subgenomes revealed by dense targeted capture linkage maps. Genome Biol Evol.

[31] Marcussen T, Heier L, Brysting AK, Oxelman B, Jakobsen KS. From gene trees to a dated allopolyploid network: insights from the angiosperm genus *Viola* (Violaceae). Syst Biol. 2015;64:84–101. 10.1093/sysbio/syu071 PMC4265142 25281848

[32] McBreen K, Lockhart PJ (2006). Reconstructing reticulate evolutionary histories of plants. Trends Plant Sci.

[33] Linder CR, Rieseberg LH (2004). Reconstructing patterns of reticulate evolution in plants. Am J Bot.

[34] Holland BR, Benthin S, Lockhart PJ, Moulton V, Huber KT (2008). Using supernetworks to distinguish hybridization from lineage-sorting. BMC Evol Biol.

[35] Brysting AK, Mathiesen C. Challenges in polyploid phylogenetic reconstruction: a case story from the arctic-alpine *Cerastium alpinum* complex. Taxon. 2011;60:333–47.

[36] Vergilino R, Markova S, Ventura M, Manca M, Dufresne F. Reticulate evolution of the *Daphnia pulex* complex as revealed by nuclear markers. Mol Ecol. 2011;20:1191–207. 10.1111/j.1365-294X.2011.05004.x 21294799

[37] Morrison DA (2014). Phylogenetic networks: a new form of multivariate data summary for data mining and exploratory data analysis. Wiley Interdiscip Rev Data Min Knowl Discov.

[38] Popp M, Erixon P, Eggens F, Oxelman B. Origin and evolution of a circumpolar polyploid species complex in *Silene* (Caryophyllaceae) inferred from low copy nuclear RNA polymerase introns, rDNA, and chloroplast DNA. Syst Bot. 2005;30:302–13.

[39] Mort ME, Crawford DJ (2004). The continuing search: low-copy nuclear sequences for lower-level plant molecular phylogenetic studies. Taxon.

[40] Duarte JM, Wall PK, Edger PP, Landherr LL, Ma H, Pires JC, et al. Identification of shared single copy nuclear genes in *Arabidopsis, Populus, Vitis *and *Oryza *and their phylogenetic utility across various taxonomic levels. BMC Evol Biol. 2010;10:61. 10.1186/1471-2148-10-61 PMC2848037 20181251

[41] Marcussen T, Jakobsen KS, Danihelka J, Ballard HE, Blaxland K, Brysting AK, et al. Inferring species networks from gene trees in high-polyploid North American and Hawaiian violets (*Viola*, Violaceae). Syst Biol. 2012;61:107–26. 10.1093/sysbio/syr096 PMC3243738 21918178

[42] McCormack JE, Hird SM, Zellmer AJ, Carstens BC, Brumfield RT (2013). Applications of next-generation sequencing to phylogeography and phylogenetics. Mol Phylogenet Evol.

[43] Mandel JR, Dikow RB, Funk VA (2015). Using phylogenomics to resolve mega-families: an example from Compositae. J Syst Evol.

[44] Nicholls JA, Pennington RT, Koenen EJM, Hughes CE, Hearn J, Bunnefeld L, et al. Using targeted enrichment of nuclear genes to increase phylogenetic resolution in the neotropical rain forest genus *Inga *(Leguminosae: Mimosoideae). Front Plant Sci. 2015;6:710. 10.3389/fpls.2015.00710 PMC4584976 26442024

[45] Griffin PC, Robin C, Hoffmann AA (2011). A next-generation sequencing method for overcoming the multiple gene copy problem in polyploid phylogenetics, applied to *Poa* grasses. BMC Biol.

[46] Browning SR, Browning BL (2011). Haplotype phasing: existing methods and new developments. Nat Rev Genet.

[47] Li H. A statistical framework for SNP calling, mutation discovery, association mapping and population genetical parameter estimation from sequencing data. Bioinformatics. 2011;27:2987–93. 10.1093/bioinformatics/btr509 PMC3198575 21903627

[48] Sasaki E, Sugino RP, Innan H. The linkage method: a novel approach for SNP detection and haplotype reconstruction from a single diploid individual using next-generation sequence data. Mol Biol Evol. 2013;30:2187-96. 10.1093/molbev/mst103 23728796

[49] Zhang Y (2013). A dynamic Bayesian Markov model for phasing and characterizing haplotypes in next-generation sequencing. Bioinformatics.

[50] Hedderson TA, Chapman RL, Rootes WL (1996). Phylogenetic relationships of bryophytes inferred from nuclear-encoded rRNA gene sequences. Plant Syst Evol.

[51] Shimizu-Inatsugi R, Lihová J, Iwanaga H, Kudoh H, Marhold K, Savolainen O (2009). The allopolyploid *Arabidopsis kamchatica* originated from multiple individuals of *Arabidopsis lyrata* and *Arabidopsis halleri*. Mol Ecol.

[52] Okuyama Y, Tanabe AS, Kato M. Entangling ancient allotetraploidization in Asian *Mitella*: an integrated approach for multilocus combinations. Mol Biol Evol. 2012;29:429–39. 10.1093/molbev/msr236 21940642

[53] Petri A, Oxelman B. Phylogenetic relationships within *Silene* (Caryophyllaceae) section *Physolychnis*. Taxon. 2011;60:953–68.

[54] Fan X, Sha L-N, Zeng J, Kang H-Y, Zhang H-Q, Wang X-L, et al. Evolutionary dynamics of the *Pgk1* gene in the polyploid genus *Kengyilia* (Triticeae: Poaceae) and its diploid relatives. PLoS One. 2012;7:e31122. 10.1371/journal.pone.0031122 PMC3282717 22363562

[55] Scheen A-C, Pfeil BE, Petri A, Heidari N, Nylinder S, Oxelman B (2012). Use of allele-specific sequencing primers is an efficient alternative to PCR subcloning of low-copy nuclear genes. Mol Ecol Resour.

[56] Straub SCK, Doyle JJ. Molecular phylogenetics of *Amorpha* (Fabaceae): an evaluation of monophyly, species relationships, and polyploid origins. Mol Phylogenet Evol. 2014;76:49–66. 10.1016/j.ympev.2014.02.025 24631856

[57] Triplett JK, Wang Y, Zhong J, Kellogg EA. Five nuclear loci resolve the polyploid history of switchgrass (*Panicum virgatum *L.) and relatives. PLoS One. 2012;7:e38702. 10.1371/journal.pone.0038702 PMC3377691 22719924

[58] Kraytsberg Y, Khrapko K (2005). Single-molecule PCR: an artifact-free PCR approach for the analysis of somatic mutations. Expert Rev Mol Diagn.

[59] Hori K, Tono A, Fujimoto K, Kato J, Ebihara A, Watano Y, et al. Reticulate evolution in the apogamous *Dryopteris varia *complex (Dryopteridaceae, subg. *Erythrovariae*, sec. *Variae*) complex and its related sexual species in Japan. J Plant Res. 2014;127:661–84. 10.1007/s10265-014-0652-0 25064510

[60] Cronn R, Knaus BJ, Liston A, Maughan PJ, Parks M, Syring JV (2012). Targeted enrichment strategies for next-generation plant biology. Am J Bot.

[61] Amini S, Pushkarev D, Christiansen L, Kostem E, Royce T, Turk C (2014). Haplotype-resolved whole-genome sequencing by contiguity-preserving transposition and combinatorial indexing. Nat Genet.

[62] Bansal V, Bafna V (2008). HapCUT: an efficient and accurate algorithm for the haplotype assembly problem. Bioinformatics.

[63] DePristo MA, Banks E, Poplin R, Garimella KV, Maguire JR, Hartl C (2011). A framework for variation discovery and genotyping using next-generation DNA sequencing data. Nat Genet.

[64] Aguiar D, Istrail S (2013). Haplotype assembly in polyploid genomes and identical by descent shared tracts. Bioinformatics.

[65] Berger E, Yorukoglu D, Peng J, Berger B. HapTree: a novel Bayesian framework for single individual polyplotyping using NGS data. PLoS Comput Biol. 2014;10:e1003502. 10.1371/journal.pcbi.1003502 PMC3967924 24675685

[66] Das S, Vikalo H (2015). SDhaP: haplotype assembly for diploids and polyploids via semi-definite programming. BMC Genomics.

[67] Weitemier K, Straub SCK, Cronn RC, Fishbein M, Schmickl R, McDonnell A, et al. Hyb-Seq: combining target enrichment and genome skimming for plant phylogenomics. Appl Plant Sci. 2014;2:1400042 10.3732/apps.1400042 PMC4162667 25225629

[68] Fortune PM, Pourtau N, Viron N, Ainouche ML. Molecular phylogeny and reticulate origins of the polyploid *Bromus* species from section *Genea *(Poaceae). Am J Bot. 2008;95:454–64. 10.3732/ajb.95.4.454 21632370

[69] Sessa EB, Zimmer EA, Givnish TJ. Unraveling reticulate evolution in North American *Dryopteris *(Dryopteridaceae). BMC Evol Biol. 2012;12:104. 10.1186/1471-2148-12-104 PMC3509404 22748145

[70] Huber KT, Oxelman B, Lott M, Moulton V (2006). Reconstructing the evolutionary history of polyploids from multilabeled trees. Mol Biol Evol.

[71] Wendel JF, Doyle JJ. Phylogenetic incongruence: window into genome history and molecular evolution. In: DE Soltis, PS Soltis, JJ Doyle, eds. Molecular Systematics of Plants II. New York City: Springer; 1998. pp. 265–96.

[72] Degnan JH, DeGiorgio M, Bryant D, Rosenberg NA (2009). Properties of consensus methods for inferring species trees from gene trees. Syst Biol.

[73] Jin G, Nakhleh L, Snir S, Tuller T (2006). Maximum likelihood of phylogenetic networks. Bioinformatics.

[74] Kubatko LS, Carstens BC, Knowles LL (2009). STEM: species tree estimation using maximum likelihood for gene trees under coalescence. Bioinformatics.

[75] Meng C, Kubatko LS (2009). Detecting hybrid speciation in the presence of incomplete lineage sorting using gene tree incongruence: a model. Theor Popul Biol.

[76] Gerard D, Gibbs HL, Kubatko L (2011). Estimating hybridization in the presence of coalescence using phylogenetic intraspecific sampling. BMC Evol Biol.

[77] Yu Y, Barnett RM, Nakhleh L (2013). Parsimonious inference of hybridization in the presence of incomplete lineage sorting. Syst Biol.

[78] Than C, Ruths D, Nakhleh L. PhyloNet: a software package for analyzing and reconstructing reticulate evolutionary relationships. BMC Bioinformat. 2008;9:322. 10.1186/1471-2105-9-322 PMC2533029 18662388

[79] Liston A, Cronn R, Ashman T-L. *Fragaria*: a genus with deep historical roots and ripe for evolutionary and ecological insights. Am J Bot. 2014;101:1686–99. 10.3732/ajb.1400140 25326614

[80] Edwards D, Batley J, Snowdon RJ. Accessing complex crop genomes with next-generation sequencing. Theor Appl Genet. 2013;126:1–11. 10.1007/s00122-012-1964-x 22948437

[81] Njuguna W, Liston A, Cronn R, Ashman T-L, Bassil N (2013). Insights into phylogeny, sex function and age of *Fragaria* based on whole chloroplast genome sequencing. Mol Phylogenet Evol.

[82] Staudt G. Strawberry biogeography, genetics and systematics. Acta Hortic. 2009;842:71–84.

[83] Potter D, Luby JJ, Harrison RE. Phylogenetic relationships among species of *Fragaria* (Rosaceae) inferred from non-coding nuclear and chloroplast DNA sequences. Syst Bot. 2000;25:337–48.

[84] Rousseau-Gueutin M, Gaston A, Aïnouche A, Aïnouche ML, Olbricht K, Staudt G, et al. Tracking the evolutionary history of polyploidy in *Fragaria *L. (strawberry): new insights from phylogenetic analyses of low-copy nuclear genes. Mol Phylogenet Evol. 2009;51:515–30. 10.1016/j.ympev.2008.12.024 19166953

[85] DiMeglio LM, Staudt G, Yu H, Davis TM. A phylogenetic analysis of the genus *Fragaria* (strawberry) using intron-containing sequence from the *ADH*-1 gene. PLoS One. 2014;9:e102237. 10.1371/journal.pone.0102237 PMC4117466 25078607

[86] Hummer KE. A new species of *Fragaria* (Rosaceae) from Oregon. J Bot Res Inst Tex. 2012;6:9–15.

[87] Aguiar D, Istrail S (2012). HapCompass: a fast cycle basis algorithm for accurate haplotype assembly of sequence data. J Comput Biol.

[88] Kubatko LS (2009). Identifying hybridization events in the presence of coalescence via model selection. Syst Biol.

[89] Liston A. 257 nuclear genes for Rosaceae phylogenomics. 2015. 10.6084/m9.figshare.1060394. Accessed 29 Jul 2017.

[90] Kent WJ (2002). BLAT--the BLAST-like alignment tool. Genome Res.

[91] Kamneva OK. Assembling haplotypes from NGS data for di- and polyploids: online tutorial. 2017. http://web.stanford.edu/~okamneva/haplotype_assembly.html. Accessed 29 Jul 2017.

[92] Shulaev V, Sargent DJ, Crowhurst RN, Mockler TC, Folkerts O, Delcher AL (2011). The genome of woodland strawberry (*Fragaria vesca*). Nat Genet.

[93] Li H, Handsaker B, Wysoker A, Fennell T, Ruan J, Homer N, et al. The sequence alignment/map format and SAMtools. Bioinformatics. 2009;25:2078–9. 10.1093/bioinformatics/btp352 PMC2723002 19505943

[94] Bolger AM, Lohse M, Usadel B. Trimmomatic: a flexible trimmer for Illumina sequence data. Bioinformatics. 2014;30:2114–20. 10.1093/bioinformatics/btu170 PMC4103590 24695404

[95] Li H, Durbin R. Fast and accurate short read alignment with Burrows-Wheeler transform. Bioinformatics. 2009;25:1754–60. 10.1093/bioinformatics/btp324 PMC2705234 19451168

[96] Kamneva OK. Phylogeny reconstruction using haplotype and consensus sequences from several Fragaria species: online tutorial. 2017. http://web.stanford.edu/~okamneva/phylogeny_fragaria.html. Accessed 29 Jul 2017.

[97] Katoh K, Standley DM (2013). MAFFT multiple sequence alignment software version 7: improvements in performance and usability. Mol Biol Evol.

[98] Guindon S, Dufayard J-F, Lefort V, Anisimova M, Hordijk W, Gascuel O (2010). New algorithms and methods to estimate maximum-likelihood phylogenies: assessing the performance of PhyML 3.0. Syst Biol.

[99] Darriba D, Taboada GL, Doallo R, Posada D (2012). jModelTest 2: more models, new heuristics and parallel computing. Nat Methods.

[100] Shimodaira H, Hasegawa M. Multiple comparisons of log-likelihoods with applications to phylogenetic inference. Mol Biol Evol. 1999;16:1114-6.

[101] Shimodaira H, Hasegawa M. CONSEL: for assessing the confidence of phylogenetic tree selection. Bioinformatics. 2001;17:1246–7. 10.1093/bioinformatics/17.12.1246 11751242

[102] Mirarab S, Reaz R, Bayzid MS, Zimmermann T, Swenson MS, Warnow T. ASTRAL: genome-scale coalescent-based species tree estimation. Bioinformatics. 2014;30:i541–8. 10.1093/bioinformatics/btu462 PMC4147915 25161245

[103] Kamneva OK. Finding and testing hybridization events in Fragaria species history: online tutorial. 2017. http://web.stanford.edu/~okamneva/testing_hybridizations.html. Accessed 29 Jul 2017.

[104] Lott M, Spillner A, Huber KT, Moulton V (2009). PADRE: a package for analyzing and displaying reticulate evolution. Bioinformatics.

[105] Huson DH, Scornavacca C. Dendroscope 3: an interactive tool for rooted phylogenetic trees and networks. Syst Biol. 2012;61:1061–7. 10.1093/sysbio/sys062 22780991

[106] Meacham CA. Theoretical and computational considerations of the compatibility of qualitative taxonomic characters. In: J Felsenstein, ed. Numerical Taxonomy. Heidelberg: Springer; 1983. pp. 304–14.

[107] Huson DH, Rupp R. Summarizing multiple gene trees using cluster networks. In: KA Crandall, J Lagergren, eds. Algorithms in Bioinformatics. Heidelberg: Springer; 2008. pp. 296–305.

[108] Hummer KE, Bassil N, Njuguna W. Fragaria. In: C Kole, ed. Wild Crop Relatives: Genomic and Breeding Resources, Temperate Fruits. New York City: Springer; 2011. pp. 17-44.

[109] Göker M, Grimm GW (2008). General functions to transform associate data to host data, and their use in phylogenetic inference from sequences with intra-individual variability. BMC Evol Biol.

[110] Joly S, Bruneau A. Incorporating allelic variation for reconstructing the evolutionary history of organisms from multiple genes: an example from *Rosa* in North America. Syst Biol. 2006;55:623–36. 10.1080/10635150600863109 16969938

[111] Potts AJ, Hedderson TA, Grimm GW (2014). Constructing phylogenies in the presence of intra-individual site polymorphisms (2ISPs) with a focus on the nuclear ribosomal cistron. Syst Biol.

[112] Joly S, Bryant D, Lockhart PJ (2015). Flexible methods for estimating genetic distances from single nucleotide polymorphisms. Methods Ecol Evol.

[113] Bertrand YJK, Scheen A-C, Marcussen T, Pfeil BE, de Sousa F, Oxelman B. Assignment of homoeologs to parental genomes in allopolyploids for species tree inference, with an example from *Fumaria* (Papaveraceae). Syst Biol. 2015;64:448–71. 10.1093/sysbio/syv004 25604357

[114] Yu Y, Dong J, Liu KJ, Nakhleh L. Maximum likelihood inference of reticulate evolutionary histories. Proc Natl Acad Sci U S A. 2014;111:16448–53. 10.1073/pnas.1407950111 PMC4246314 25368173

[115] Roux C, Pannell JR (2015). Inferring the mode of origin of polyploid species from next-generation sequence data. Mol Ecol.

[116] Kubatko L, Chifman J. An invariants-based method for efficient identification of hybrid species from large-scale genomic data. 2015; doi: https://doi.org/10.1101/034348. 10.1186/s12862-019-1439-7 PMC6543680 31146685

[117] Kamneva OK, Rosenberg NA. Simulation-based evaluation of hybridization network reconstruction methods in the presence of incomplete lineage sorting. Evol Bioinf. 2017;13:1176934317691935. 10.1177/1176934317691935 PMC5395256 28469378

[118] Grover CE, Gallagher JP, Jareczek JJ, Page JT, Udall JA, Gore MA (2015). Re-evaluating the phylogeny of allopolyploid *Gossypium* L. Mol Phylogenet Evol.

[119] Snyder MW, Adey A, Kitzman JO, Shendure J (2015). Haplotype-resolved genome sequencing: experimental methods and applications. Nat Rev Genet.

[120] Patterson M, Marschall T, Pisanti N, van Iersel L, Stougie L, Klau GW, et al. WhatsHap: haplotype assembly for future-generation sequencing reads. In: R Sharan, ed. Research in Computational Molecular Biology. New York City: Springer; 2014. pp. 237–49. 10.1089/cmb.2014.0157 25658651

[121] Halldórsson BV, Aguiar D, Istrail S. Haplotype phasing by multi-assembly of shared haplotypes: phase-dependent interactions between rare variants. Pac Symp Biocomput. 2011:16:88–99. 10.1142/9789814335058_0010 21121036

[122] Staudt G, Dickore W. Notes on Asiatic *Fragaria* species: *Fragaria pentaphylla* Losinsk. and *Fragaria tibetica* spec. nov. Bot Jahrb. 2001;123:341–54.

[123] Staudt G. Notes on Asiatic *Fragaria* species: III. *Fragaria orientalis* Losinsk. and *Fragaria mandshurica* spec. nov. Bot Jahrb. 2003;124:397–419.

