# Supplementary material for: Evaluating allopolyploid origins in strawberries (Fragaria) using haplotypes generated from target capture sequencing
Source: BMC Evol Biol. 2017 Aug 4;17:180. doi: 10.1186/s12862-017-1019-7 (PMC5543553; doi:10.1186/s12862-017-1019-7)
Supplement: Supplementary file 4 — Supplementary materials. Figs. S1-S11. (PDF 3514 kb) [file 12862_2017_1019_MOESM4_ESM.pdf]

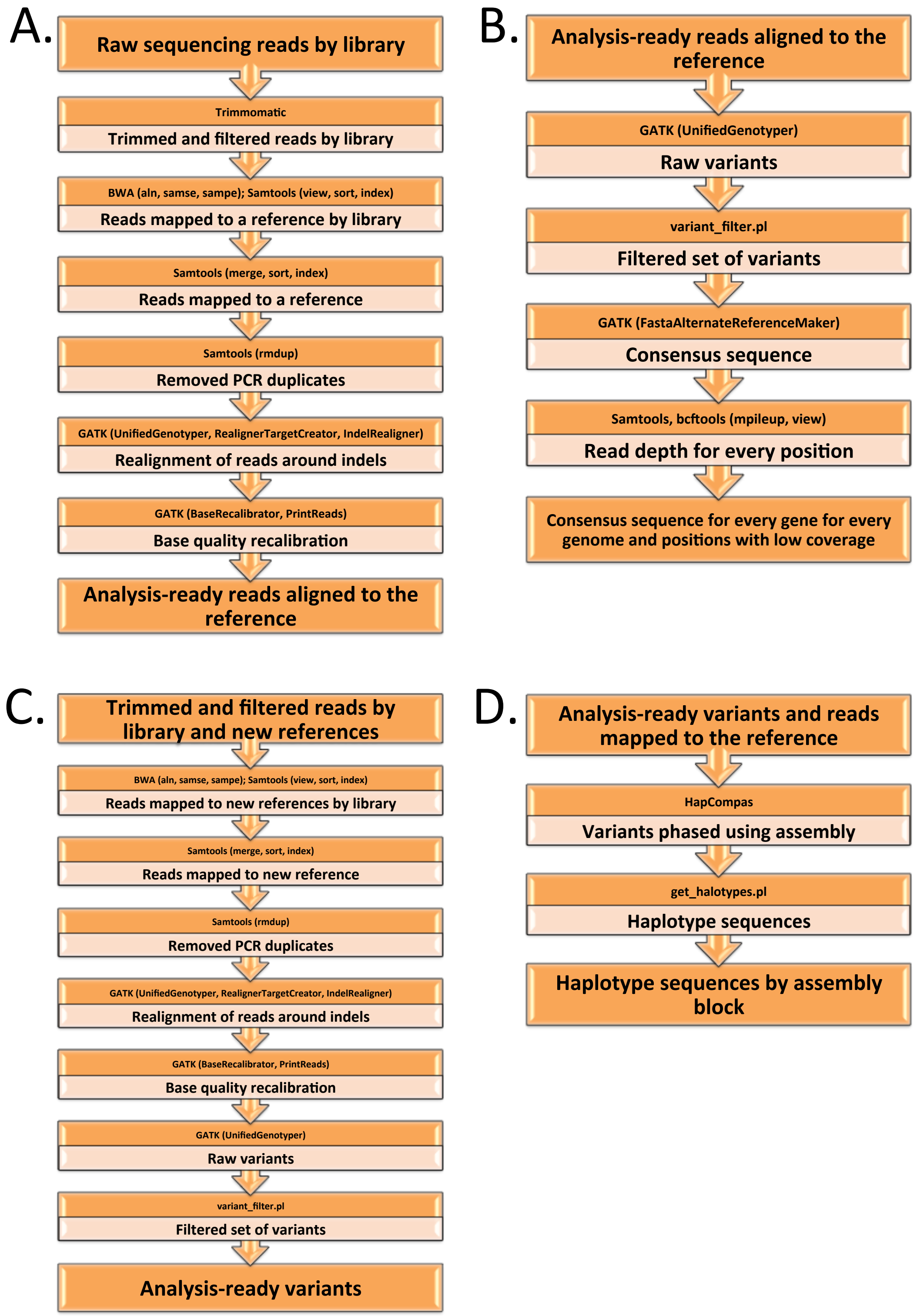

Figure S1. Haplotype assembly pipeline (Step 2 in Figure 1). Processing proceeds from step A to B to C to D, generating haplotype sequences by assembly blocks and consensus sequences for covered regions of the reference genome. These sequences are subsequently used in Step 3 in Figure 1.

A.

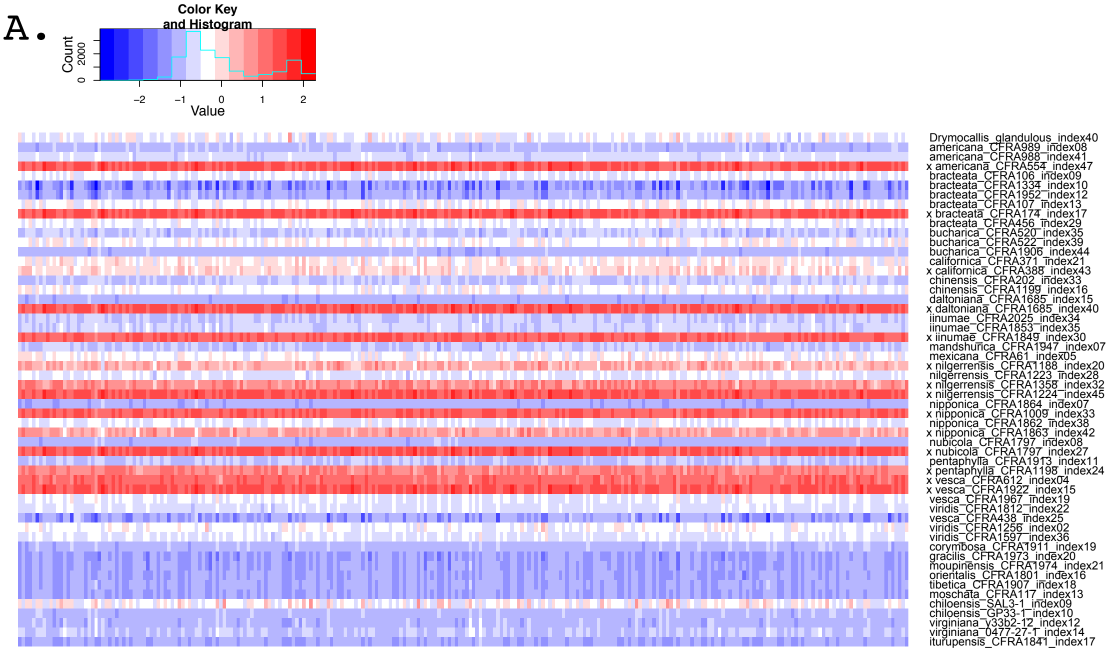

B.

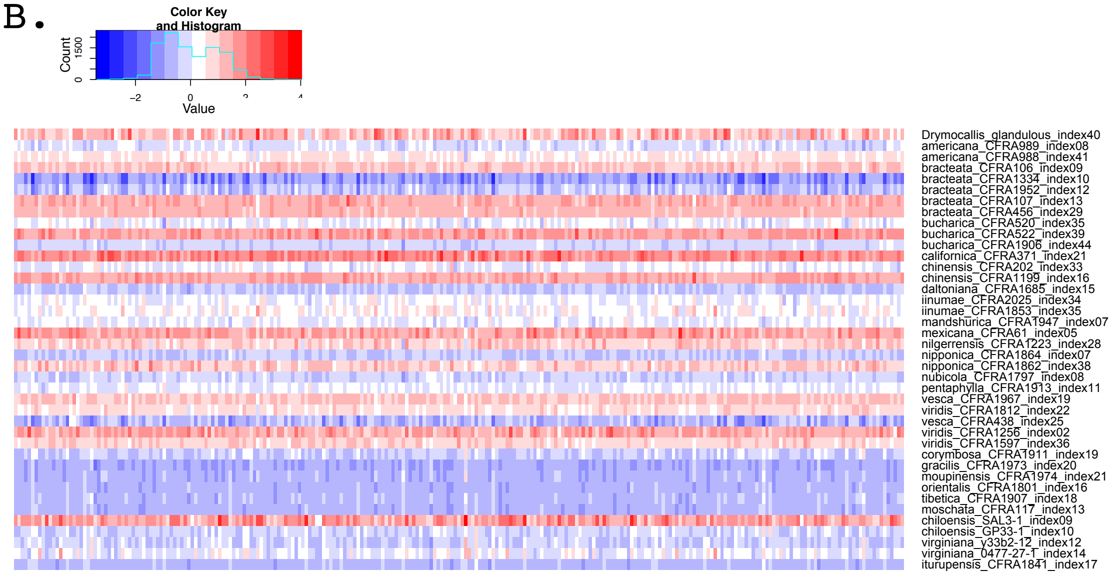

Figure S2. Heatmap depicting the fraction of gene length not covered by sequencing. The threshold for depth of coverage was set at 3 reads per position. The fraction depicted is normalized and standardized across all the genomes by gene. Colors represent individual values as described in the color key. Histogram in the color key indicates the distribution of the colors in the heatmap.

A. All 54 genomes. Excluded genomes that had average normalized, standardized fractions of genes uncovered over 0.25 when averaged across all the genes, are marked by “x” before their names.

B. The 40 retained genomes only.

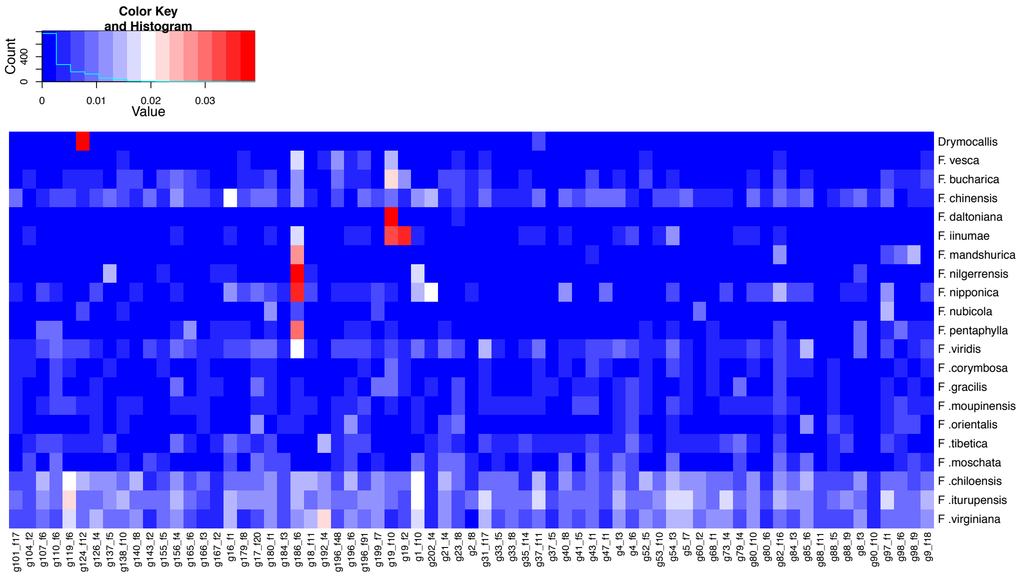

Figure S3. Distance between haplotypes calculated under the Kimura (1980) substitution model and averaged across all the comparisons for a given fragment and species. Fragments are in columns and are marked with gene number and fragment number; species are in rows. Colors represent individual values as described in the color key. The histogram in the color key indicates the distribution of the colors in the heatmap.

```
Input:
map = matrix, mapping positions of consensus sequences from different organisms onto each other, generated from the multiple sequence alignment
blocks = for each individual it is a set of blocks where haplotypes were assembled, positions correspond to position in consensus reference sequence

Output:
haplotype_fragmets = regions with continuous haplotype assembly across all of the genomes


blocks_new_borders = obtain new positions of haplotype assembly blocks by individual using map
total_positions = number of columns in the map
haplotype_fragmets = empty list

set position to 1
while position <= total_positions
            haplotypes_list_by_individual = obtain list of haplotype assembly blocks covering position by individuals using blocks_new_borders

            right_borders = empty list
            for each individual in map
                        right_border = find haplotype from corresponding unit of haplotypes_list_by_individual which has largest end position
                        append right_border to right_borders
                        remove haplotype blocks which have end before right_border from corresponding unit of haplotypes_list_by_individual
            right_border = minimum of right_borders

            left_borders = empty list
            for each individual in map
                        left_border = first position of haplotype assembly block from corresponding unit of haplotypes_list_by_individual
                        append left_border to left_borders

            left_border = maximum of left_borders

            append right_border, left_border pair to haplotype_fragmets
            set position to right_border+1


set position to 1
while position <= total_positions
            haplotypes_list_by_individual = obtain list of haplotype assembly blocks covering position by individuals using blocks_new_borders

            left_borders = empty list
            for each individual in map
                        left_border = find haplotype from corresponding unit of haplotypes_list_by_individual which has smallest end position
                        append left_border to left_borders
                        remove haplotype blocks which have start after left_border from corresponding unit of haplotypes_list_by_individual
            left_border = maximum of left_borders


            right_borders = empty list
            for each individual in map
                        right_border = of haplotype assembly block from corresponding unit of haplotypes_list_by_individual
                        append right_border to right_borders
            right_border = minimum of right_borders

            append right_border, left_border pair to haplotype_fragmets
            set position to right_border+1
```

Figure S4. Outline of a procedure for cutting out sequence fragments for phylogenetic analysis. The procedure was applied in Step 3 in Figure 1.

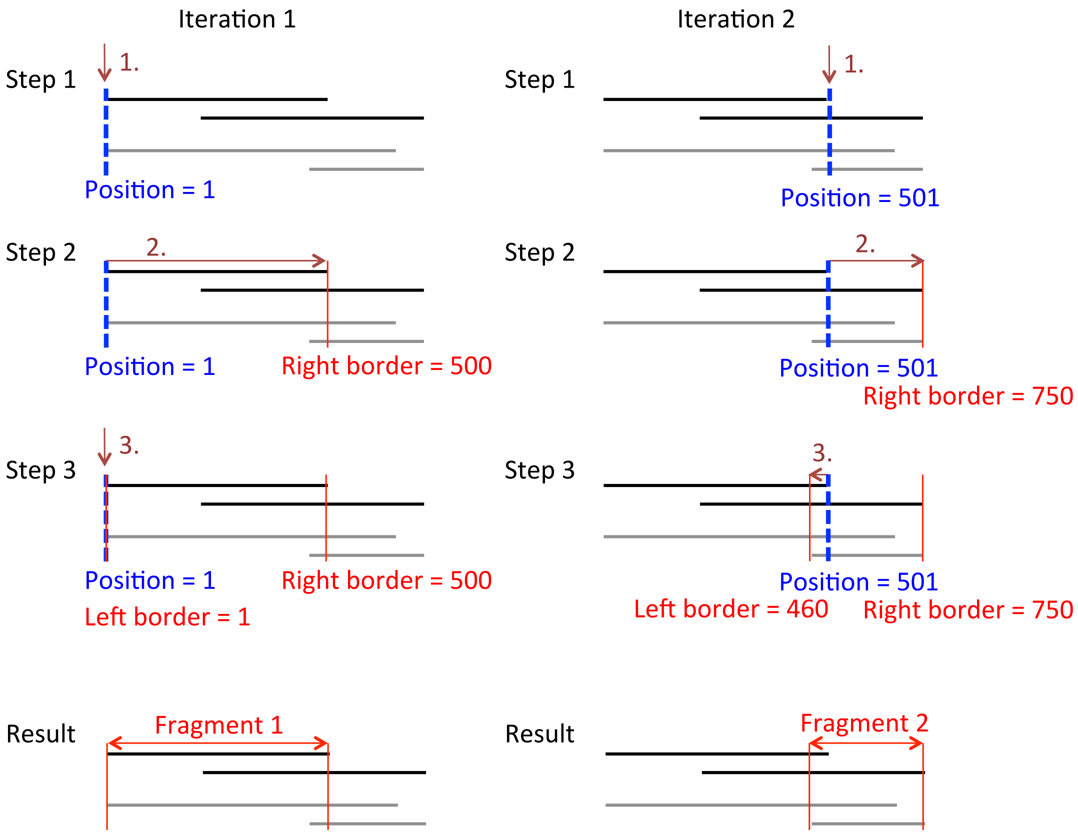

Figure S5. Cartoon representation of a procedure for cutting out sequence fragments for phylogenetic analysis. The procedure was applied in Step 3 in Figure 1.

Here two individuals are represented by haplotypes (grey and black horizontal lines). For each of them given gene was assembled in two haplotype blocks between which phase could not be determined. Procedure would follow 3 steps and would reach the end of the gene in 2 iterations yielding 2 fragments of continuous haplotype assembly across two individuals.

Input:
List trees = (T_1, T_2, ... T_1) of MUL-trees on taxa set M.
r in [0, 1], minimal fraction of trees to support cluster for it to be included into final species relationship graph even if it is in conflict with other clusters and would produce reticulation node.
t in [0, 1], minimal fraction of trees to contain cluster for it to be included into final species relationship graph only if it is not in conflict with other clusters and would produce a tree node.
b in [0, 1], minimal bootstrap support of clade in gene tree in order for a clade to contribute towards clusters supported by gene tree.

Output:
Species network

```
networkFromMULTrees(trees, r, t, b):
            set clusters as empty list of elements
            for tree in trees:
                        clusters_i = clustersFromMULTree(tree, b)
                        unique_clusters_i = unique clusters_i
                        append unique_clusters_i to clusters


            unique_clusters = unique clusters
            set l to length of list trees
            set clusters_to_include to empty list of lists
            set counts to empty list of elements
            for cluster in unique_clusters:
                        set count to number of times cluster appears in clusters
                        append count to counts
                        if count is larger or equal to r*l:
                                    append cluster to clusters_to_include

            set count_ordered_clusters to list of clusters ordered by descending order of counts
            for cluster in clusters:
                        if cluster is not in clusters_to_include:
                                    if not isConflicting(cluster, clusters_to_include):
                                                append cluster to clusters_to_include



            append species names to clusters_to_include
            sorted_clusters_to_include = sort clusters_to_include by size of cluster in ascending order

            set network to empty 0,2 matrix that will contain edges as (parent, child) pairs
            set parents to empty list of lists that will contain parent information for every node of network
            set node_names to empty list that will contain names of network nodes arranged in the order of nodes
            set current_node to 0

            append (1, 2) edge to network
            append "root" and first cluster from clusters to node_names
            set current_node to 2
            append empty list and first cluster from clusters to parents

            starting from the second cluster for every cluster in clusters
                        increment current_node by 1
                        append empty list to parents
                        set cluster_parents to empty list
                        set organisms_in_cluster to species in cluster
                        check all node_name already in node_names, from last node up to second one as first one is root, track node number (n)
                                    if no cluster_parents are equal to node_name
                                                set organisms_in_node to to species in node_name
                                                if all organisms_in_cluster are in organisms_in_node
                                                            append new edge (n, current_node) to network
                                                            append node_name and parents of node_name to last element of parents
                                                            set cluster_parents to all clusters in the last element of parents
                                                            if current_node is not yet in node_names
                                                                        append cluster to node_names



                        if is.hybridization(network, current_node)
                                    append new edge (current_node, current_node+1) to network
                                    set parents of current_node+1 to parents of current_node
                                    increment current_node by 1
                                    append cluster to node_names



            return network and node_names


clusters_from_MUL_tree(tree, b):
            set clusters to empty list of lists
            for each internal node of tree
                        if node_label is larger or equal to b
                                    set labels to list of tip names of clade descendant from node
                                    set sorted_unique_labels to list of sorted, unique labels
                                    append sorted_unique_labels to clusters
            return clusters



isConflicting(cluster_to_test, clusters_to_include):
            set result to FALSE
            for each accepted_cluster in clusters_to_include
                        if organisms in accepted_cluster and organisms in cluster_to_test overlap
                                    if there are unique organisms in both organisms in accepted_cluster and organisms in cluster_to_test
                                                set result to TRUE

            return result


is.hybridization(network, node):
            set node_parents to the list of node parents
            if length of node_parents equal to 1
                        return FALSE
            else
                        return TRUE
```

Figure S6. Outline of a procedure for building consensus species networks from gene trees. The procedure was applied in Step 5 in Figure 1.

Support for reticulation nodes:

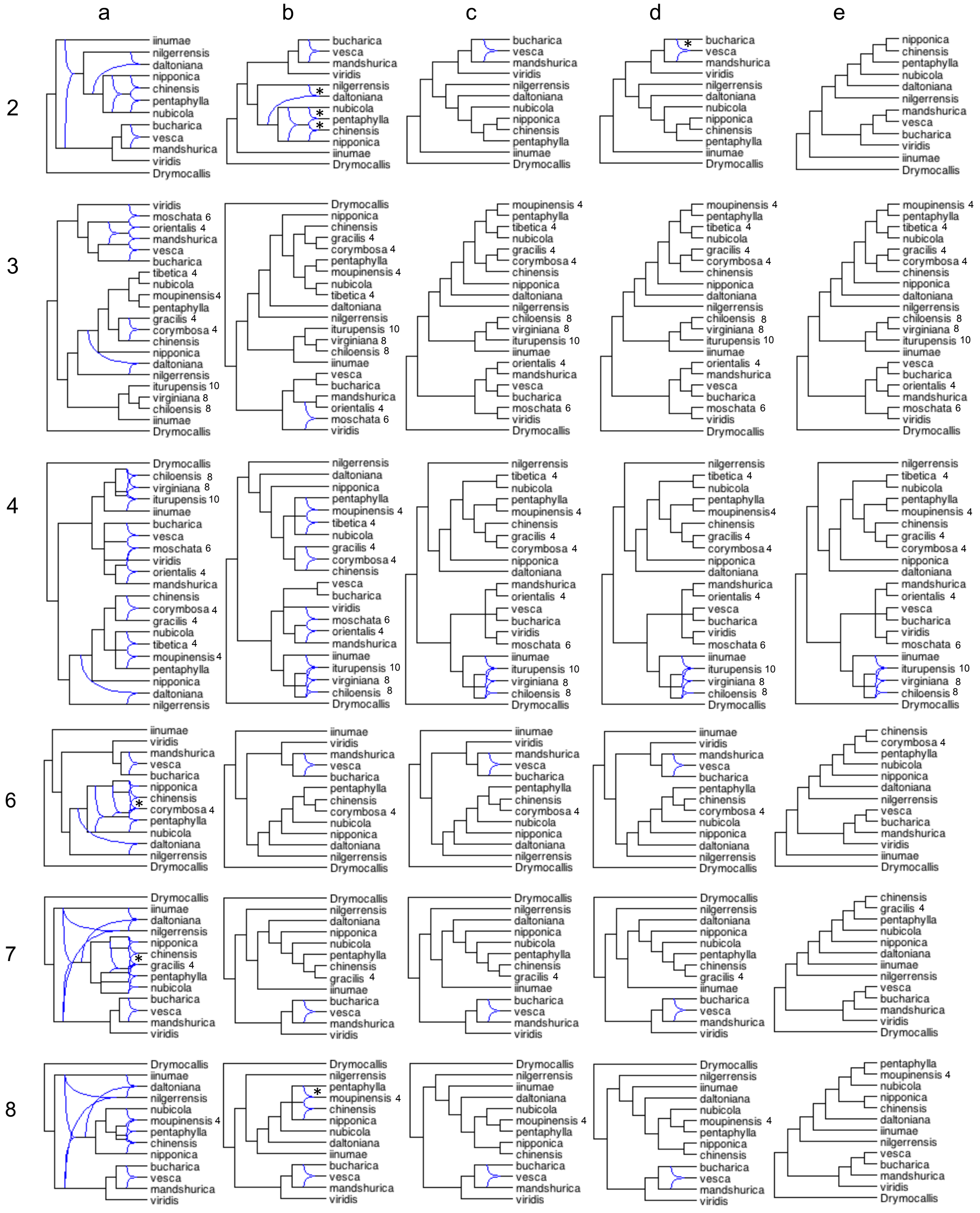

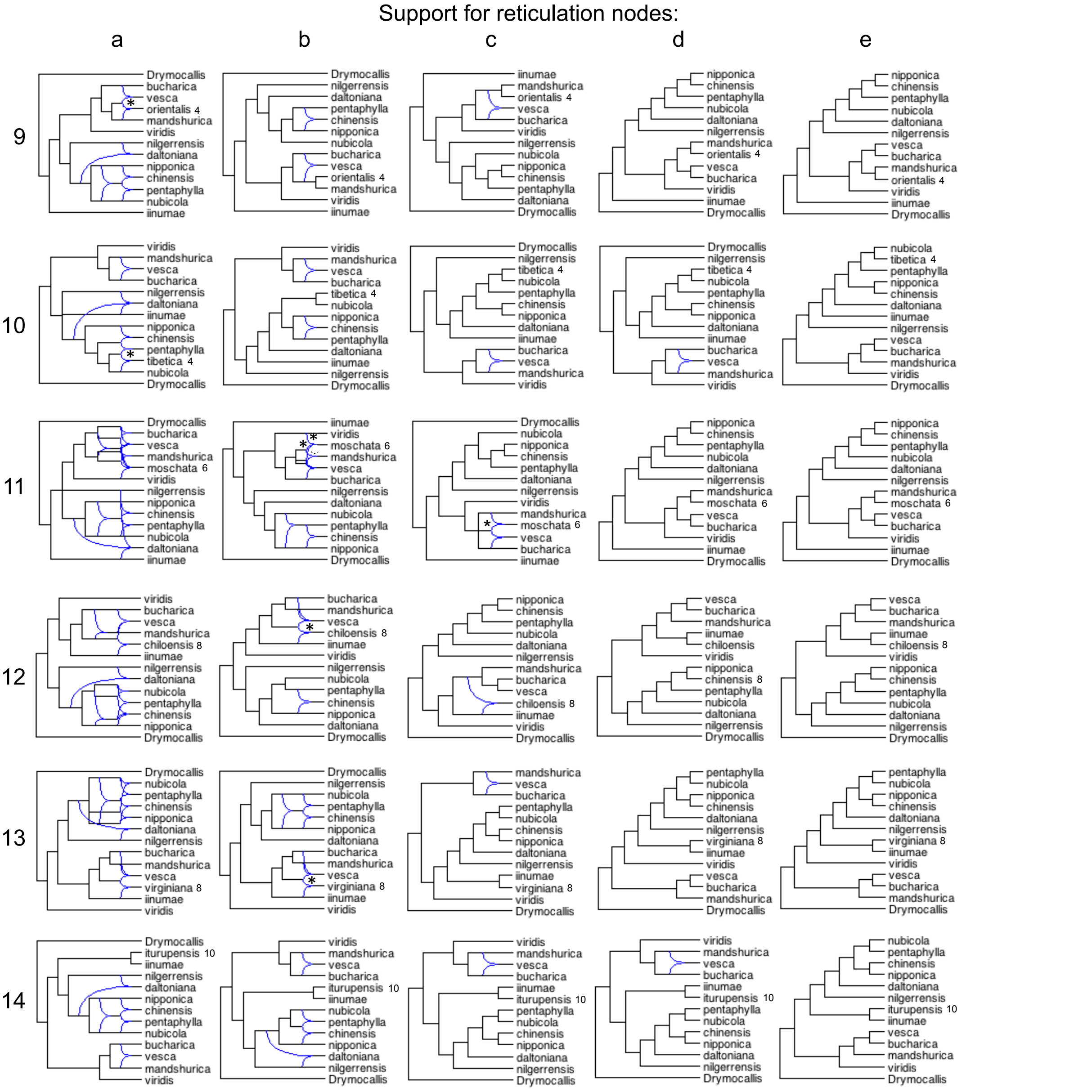

Figure S7. Consensus cluster networks for every set of taxa, constructed using all the fragments passing the SH test against 100 random trees. The percent indicates the minimum support required for a cluster to be included in the procedure for identifying putative hybridizations. (a) 15%. (b) 20%. (c) 30%. (d) 40%. (e) 50%. The asterisks indicate potential hybridization events with diploid progenitors involved in homoploid speciation (dataset 2) or the origins of a polyploid species (datasets 6-14). The asterisks are placed at the highest level of support for that hybridization event and indicate only those cases that were tested in STEMhy and PhyloNet (Tables 1 and 2).

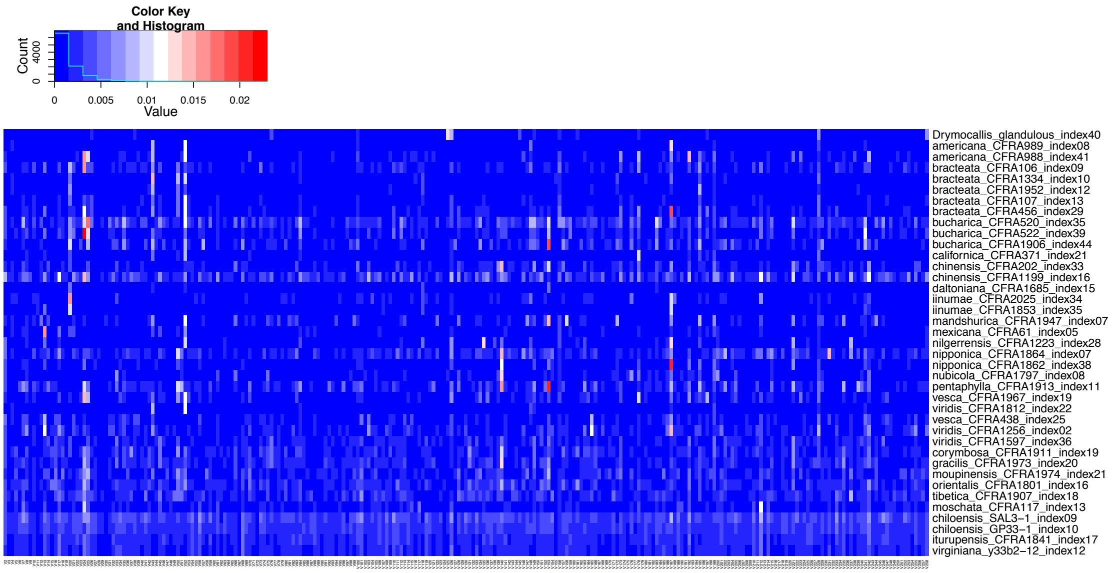

Figure S8. The fraction of all sites that were variable within covered regions of the gene divided by ploidy level of the organism. Different genes are shown in columns and individuals are in rows. Colors represent individual values as described in the color key. The histogram in the color key indicates the distribution of the colors in the heatmap.

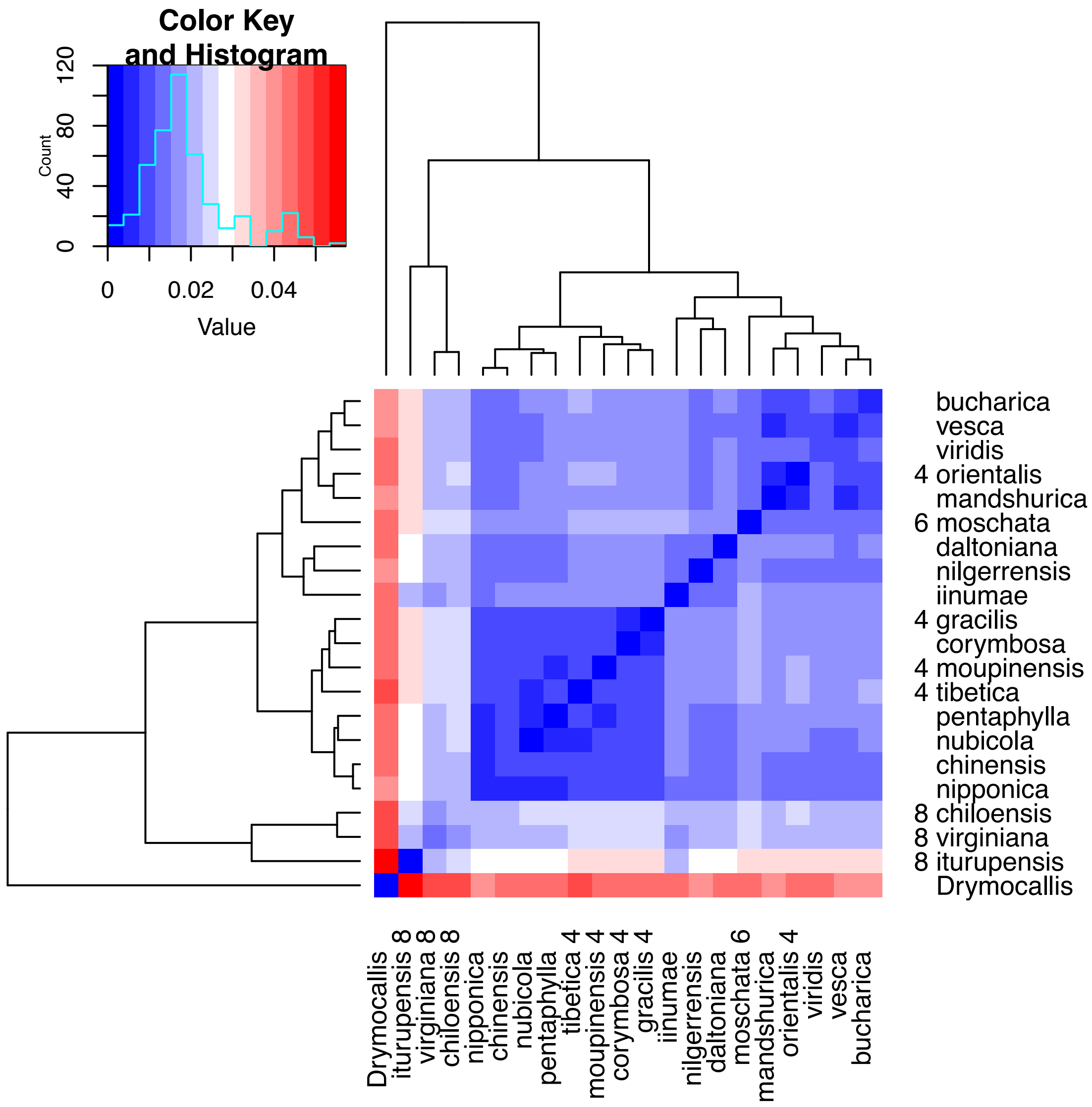

Figure S9. Distances between consensus sequences calculated under the Kimura (1980) substitution model, averaged across all the individual comparisons across species pairs and genes. Ploidy level is given for non-diploid species next to species names. Colors represent individual values as described in the color key. The histogram in the color key indicates the distribution of the colors in the heatmap.

A. Percent of sequence in longest haplotype

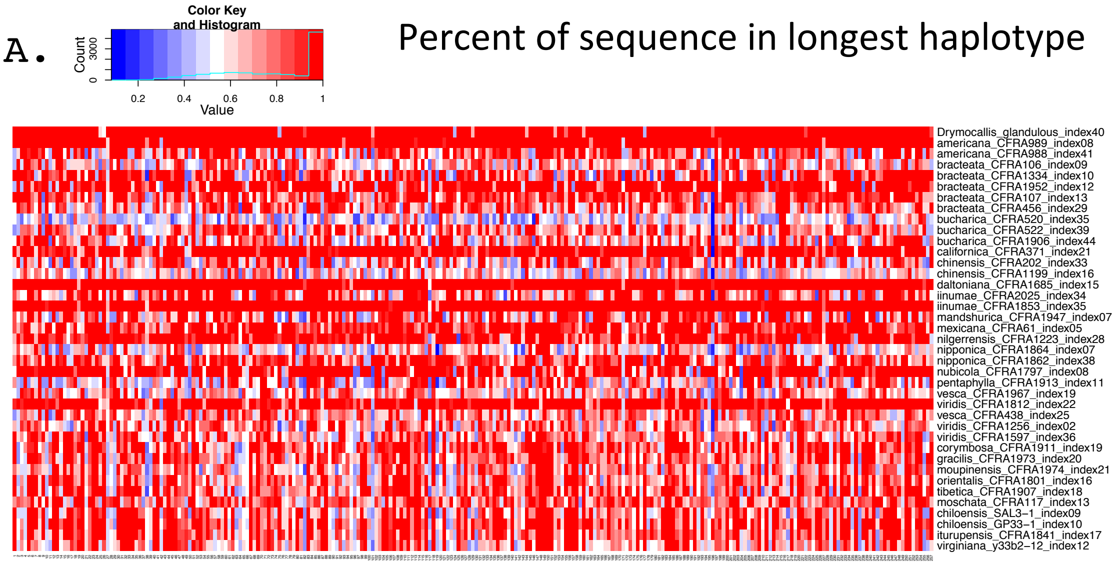

B. Percent of heterozygous positions in longest haplotype

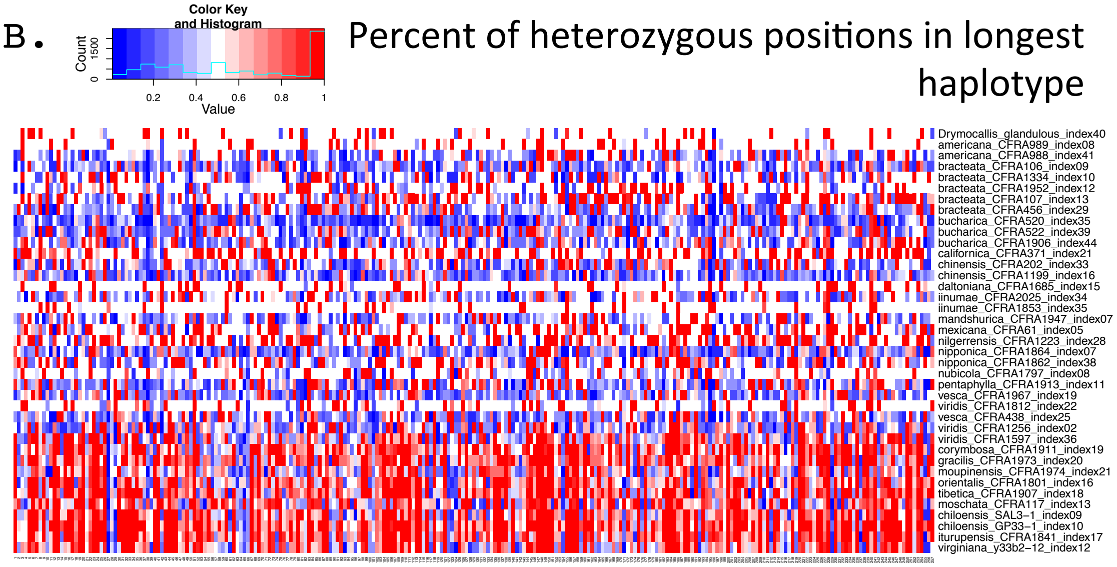

C. Percent of sequence in haplotype with largest number of heterozygous positions

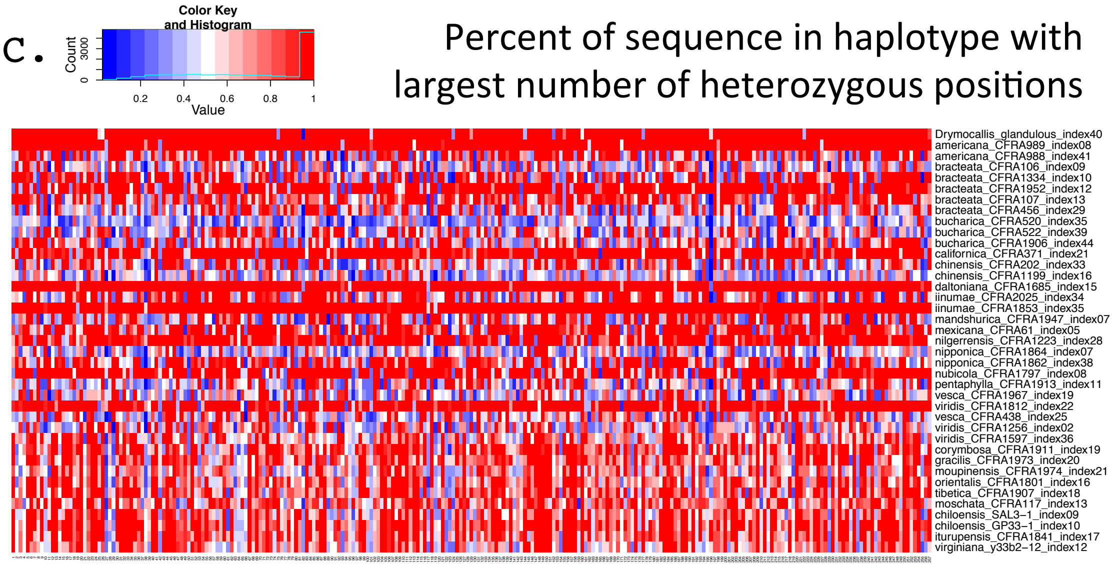

D. Percent of heterozygous positions in haplotype with largest number of heterozygous positions

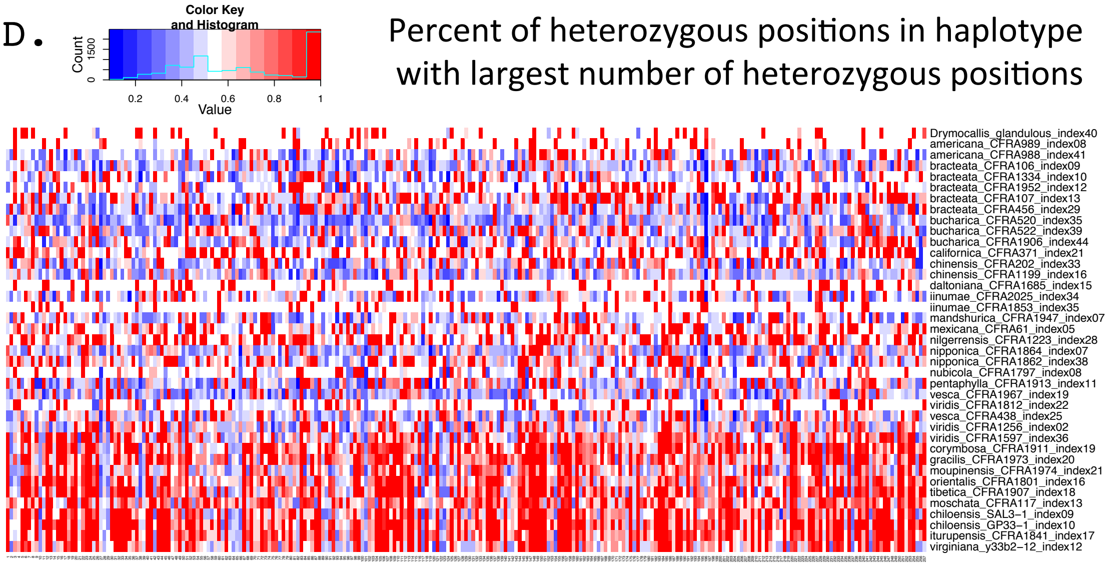

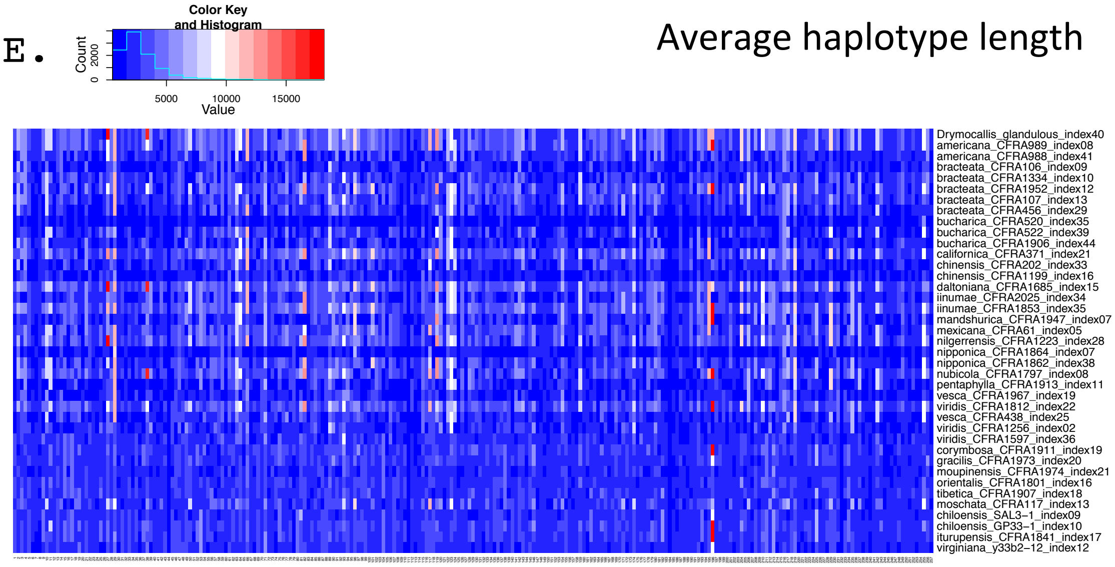

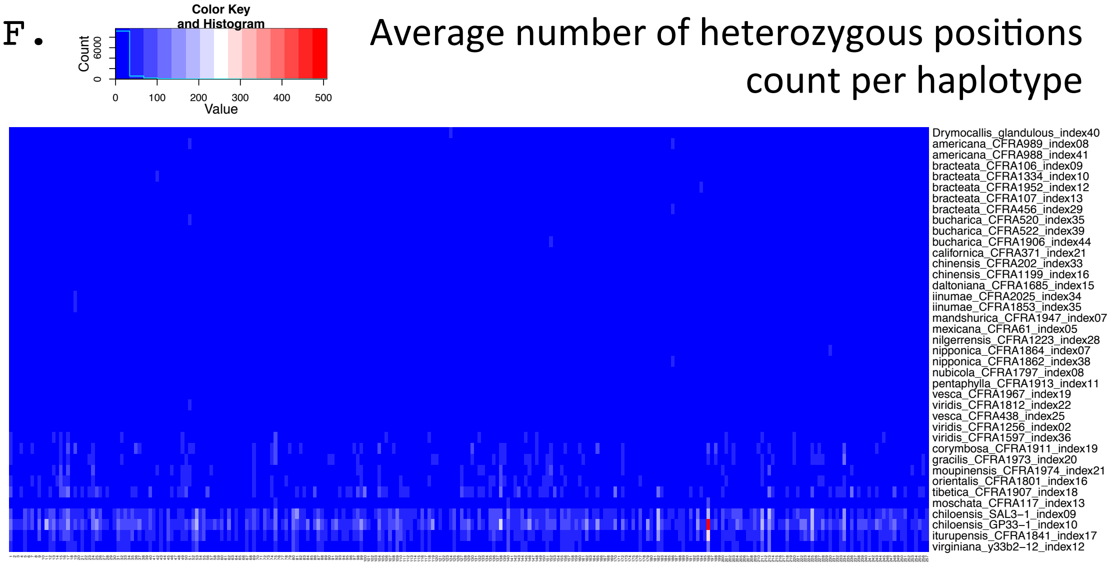

Figure S10. Results of haplotype assembly.
A. Percent of sequence in longest haplotype out of entire length of the gene covered by reads;
B. Percent of heterozygous positions in longest haplotype out of all heterozygous positions;
C. Percent of sequence in haplotype with largest number of variants out of entire length of the gene covered by reads;
D. Percent of heterozygous positions in haplotype with largest number of variants out of all heterozygous positions;
E. Average haplotype length for a given gene in a given individual;
F. Average number of heterozygous positions per haplotype for the given gene in a given individual.

Different genes are shown in columns and individuals are in rows. Colors represent individual values as described in the color key in every panel. The histogram in the color key indicates the distribution of the colors in the heatmap.

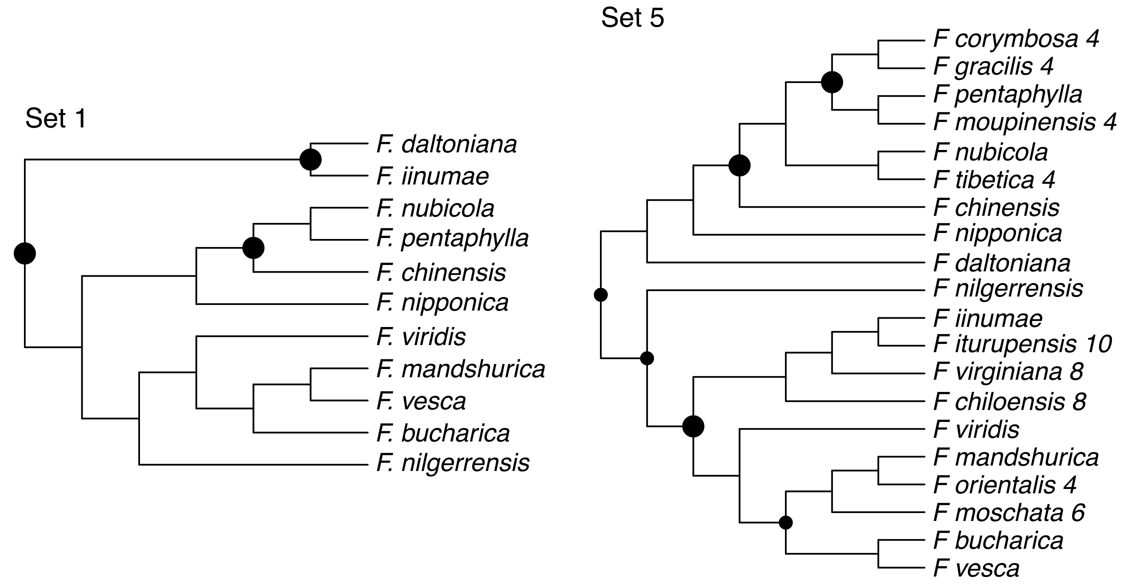

Figure S11. Species trees reconstructed using ASTRAL for two datasets.
Topologies were tested using bootstrap trees generated by PhyML. Clades observed in over 85% of bootstrap replicates are not labeled, clades observed in 70% to 85% of replicates are marked with small circles, and those observed in 70% of replicates or less are marked with large circles. Ploidy levels are shown for non-diploid species. Branch lengths are not to scale. This figure shows two datasets not included in Figure 3.
